# Whole genome sequencing and phylogenetic classification accelerate the implementation of respiratory syncytial virus genomic surveillance in Canada: a pilot study

**DOI:** 10.1128/spectrum.03142-24

**Published:** 2025-09-03

**Authors:** Ruimin Gao, Cody Buchanan, Kerry Dust, Paul Van Caeseele, Henry Wong, Calvin Sjaarda, Prameet M. Sheth, Agatha N. Jassem, Jessica Minion, Nathalie Bastien

**Affiliations:** 1 National Microbiology Laboratory, Public Health Agency of Canada, Winnipeg, Manitoba, Canada; 2 Virus Detection, Cadham Provincial Laboratory, Winnipeg, Manitoba, Canada; 3 Kingston Health Sciences Centre and Queen’s University, Kingston, Ontario, Canada; 4 British Columbia Centre for Disease Control, Provincial Health Services Authority 644858, Vancouver, British Columbia, Canada; 5 Laboratory Medicine, Saskatchewan Health Authority 7234, Regina, Saskatchewan, Canada; Victorian Infectious Diseases Reference Laboratory, Melbourne, Australia

**Keywords:** RSV, multiplex tiling PCR, whole genome sequencing, genomic surveillance, phylogeny, vaccine and antiviral drug

## Abstract

Whole genome sequencing (WGS) has emerged as a powerful tool to facilitate the study of existing and emerging infectious diseases. WGS-based genomic surveillance provides information on the genetic diversity and tracks the evolution of important viral pathogens, including respiratory syncytial virus (RSV). Multiplex tiling polymerase chain reaction (PCR) assays have been used to facilitate sequencing of a variety of pathogens in support of genomics-based surveillance initiatives. We developed, optimized, and implemented multiplex tiling PCR assays for RSVA and RSVB capable of generating near-complete genomes in the majority of contemporaneous specimens tested. A pilot data set comprising 52 RSVA and 37 RSVB genomes derived from Canadian clinical specimens during the 2022–2023 respiratory virus season was used to perform phylogenetic analyses using both near-complete genome and glycoprotein (G) sequences. Overall, the RSV phylogenetic tree built with whole genomes showed identical lineage clusters as compared to the *G* gene but was more discriminatory. Moreover, the availability of complete genomes enables the identification of a broader range of mutations. For instance, mutations identified in the fusion protein among Canadian isolates tested here, including S377N, K272M, S276N, S211N, S206I, and S209Q, could affect the efficacy of current vaccines or antiviral-based therapeutics. In conclusion, our work reinforces other recent studies demonstrating the utility of multiplex tiling PCR assays to facilitate high-throughput WGS of RSV, which is capable of supporting enhanced genomic surveillance initiatives, as well as the more comprehensive genomic analyses required to inform public health strategies for the development and usage of vaccines and antiviral drugs.

**IMPORTANCE:**

We present assays to efficiently sequence genomes of RSVA and RSVB. This enables researchers and public health agencies to acquire high-quality genomic data using rapid and cost-effective approaches. Genomic data-based comparative analysis can be used to conduct surveillance and monitor circulating isolates for efficacy of vaccines and antiviral therapeutics.

## INTRODUCTION

Respiratory syncytial virus (RSV), also called human respiratory syncytial virus (hRSV), is a common, contagious airborne viral infection that primarily affects the respiratory tract (1, 2). RSV is a negative-sense enveloped single-stranded non-segmented RNA virus belonging to the *Paramyxoviridae* family, genus *Orthopneumovirus*. This virus has a ~15.2 kb genome that encodes 11 proteins, including 3 surface glycoproteins: the fusion protein (F), attachment glycoprotein (G) and the small hydrophobic protein (SH), the RNA-dependent RNA polymerase large protein (L), nucleocapsid (N), phosphoprotein (P), transcriptional regulators (M2-1 and M2-2), matrix (M), and non-structural proteins (NS1 and NS2) (3). The F and G proteins promote the production of protective immune response, and antigenic differences in the G, F, and SH envelope proteins have led to the classification of RSV into two major antigenic groups: RSVA and RSVB. The F protein is responsible for the fusion of the viral envelope with the host cell membrane for the viral entry into the cell and is highly conserved and immunogenic, which is attractive for vaccine development. In contrast, the G protein, which is responsible for cellular attachment, is prone to frequent mutations (3–5).

RSV diagnosis is usually based on clinical symptoms, but laboratory tests such as rapid antigen tests (6, 7) and polymerase chain reaction (PCR) (8) are commonly used diagnostic methods. In recent years, whole genome sequencing (WGS) and its analysis have provided a more in-depth understanding of RSV outbreaks (9–11). By identifying and characterizing viral isolates causing outbreaks, public health authorities and institutions can implement appropriate control measures, such as isolation, contact tracing, and targeted vaccination campaigns (11). WGS-based genomic surveillance involves the systematic monitoring and analysis of viral genomes to understand their genetic diversity, track transmission patterns, and inform public health interventions (12). It has been instrumental in characterizing RSV isolates circulating globally, identifying emerging clades or subclades, and assessing their impact on disease severity and vaccine efficacy. Initially based on sequences derived from the *G* gene, five RSVA and four RSVB clades were identified, named GA1–GA5 and GB1–GB5, respectively. Nextclade (https://clades.nextstrain.org/) has now included both the G_Clade (13) and recently standardized hRSV Genotyping Consensus Consortium (RGCC) lineages for classifications (14, 15).

WGS-based genomic surveillance provides complete genetic profiles on the pathogens and enables researchers to identify specific genetic mutations, including single-nucleotide polymorphisms, insertions, deletions, and rearrangements throughout the viral genomes. Especially since the RNA-dependent replication cycle of RSV is error prone with no proofreading mechanism (16). Analysis of genetic variations and evolutionary patterns informs our understanding of how the virus evolves over time and how new isolates or variants emerge. Furthermore, genomic surveillance helps monitor for genetic changes that may affect the antigenicity of RSV isolates (13). By examining specific genomic regions associated with viral surface proteins (e.g., the G, F, and SH), researchers can identify potential variations that might affect the effectiveness of vaccines or antiviral-based therapeutics or prophylactic monoclonal antibodies (mAbs). This information informs vaccine development strategies and the selection of vaccine isolates (17).

Although there are a number of RSV vaccine candidates in the pipeline (18–20), since late 2023, only two have been approved for use in Canada for adults over 60 years of age, including RSVPreF3 (Arexvy) and RSVpreF (Abrysvo) (20, 21). Both comprise elements of the RSV F protein stabilized in its prefusion conformation (22). Additionally, there are two prophylactic RSV mAbs approved for use in Canada, including palivizumab (SYNAGIS), which is targeted against the F antigenic site II, and nirsevimab (Beyfourtus), which is targeted against the F antigenic site Ø. In Canada, the use of palivizumab has been reserved for at-risk infants and children under 2 years of age largely due to cost, limited efficacy, and the requirement of multi-dose regimen. Conversely, nirsevimab, approved for use in Canada in 2023, requires only a single dose and is being recommended for all newborns (22). Nirsevimab makes use of specifically engineered amino acid (aa) changes (M257Y/S259T/T261E) to the fragment crystallizable region of the immunoglobulin G antibody that results in an extended half-life in serum capable of providing protection with one injection for the entire RSV season (23, 24). Thus, with wider use of both vaccines and mAbs, it is critical to perform both serological and genomics-based surveillance to identify any shift in antigenicity among circulating viruses that could impact RSV vaccines and therapeutics used in Canada (20).

It is worth mentioning that the coronavirus disease 2019 pandemic has significantly influenced research activities across various fields, including those for RSV. Although several WGS-based methods for RSV have been reported previously, their strategies relied upon amplification of many individual PCR products before pooling them for sequencing (25, 26), hybridization to capture probes (27, 28), or metagenomic sequencing (29), which are time-consuming and not ideal for high-throughput routine analysis. In contrast to these methods, the multiplex tiling PCR amplification approach is more amenable to routine and high-throughput sequencing operations. Given recent approvals in Canada and abroad for vaccines targeting RSV, we and others have developed assays targeting RSVA and RSVB to support surveillance initiatives (30–32). To validate the assays described here, and to explore both the genomic diversity of RSV and carriage of important mutations within strains circulating in Canada, we conducted a pilot study comprising 89 representative clinical specimens from four different provinces, including Ontario (ON), Manitoba (MB), British Columbia (BC), and Saskatchewan (SK). Our results demonstrate that the multiplex tiling PCR approach and subsequent genomic analyses have great potential to accelerate large-scale RSV sequencing, diagnosis, and genomic surveillance in Canada. Furthermore, these multiplex tiling PCR assays enable the simultaneous amplification of the complete genome of this virus, allowing for a more comprehensive analysis of its genetic diversity and evolution with enhanced efficiency and sensitivity, which will enable researchers to better understand RSV and inform public health strategies regarding vaccine and antiviral development and usage.

## MATERIALS AND METHODS

### Reference viruses and clinical specimens

Reference RSV isolates used in this study were purchased from Cedarlane and include VR-955 (strain 9320, originally collected in 1977), VR-1803 (strain ATCC-2012-11, originally collected in 2012) and VR-1794 (strain ATCC-2012-10, originally collected in 2012). RSV-positive nasopharyngeal swabs were kindly provided by four provincial laboratories, including the British Columbia Center for Disease Control (BC), Roy Romanow Provincial Laboratory (SK), Cadham Provincial Laboratory (MB), and Kingston Health Sciences Center (ON). All specimens were collected during the 2022–2023 respiratory virus season, with the exception of four specimens from 2016 (SK), one from 2019 (MB), and two from 2021 (BC).

### Development of the multiplex tiling PCR amplification assays

#### RSVA

The initial assay was designed against a data set comprising all publicly available complete RSVA genomes (*n* = 869, accessed 22 November 2022) downloaded from the Bacterial and Viral Bioinformatics Resource Center (BVBRC) (https://www.bv-brc.org/). The genomes were clustered at 99% sequence identity using cd-hit-est (v.4.8.1) (33), resulting in 71 clusters, and the corresponding genomes representing each cluster were aligned using MAFFT (v.7.505) (34). The alignment was reordered such that the first sequence corresponded to the largest cluster with subsequent sequences representing smaller clusters. The reordered alignment was processed using PrimalScheme (v.1.3.2) (30), which was used to develop a multiplex tiling PCR scheme with default settings, except the amplicon size range was iterated in 100 nucleotide (nt) increments from 400 to 500 nt through 1,100–1,200 nt to identify a primer set with the broadest coverage across the genome. The final assay used an amplicon size range of 800–900 nt, and the RSVA isolate, SE01A-0167-V02 (GenBank accession no. MZ515773.1), was selected as the coordinate system for PrimalScheme (30). An additional 2,527 RSVA genomes were acquired from Global Initiative on Sharing All Influenza Data (GISAID) (accessed 24 November 2022) and combined with those from the BVBRC for a combined data set comprising 3,396 genomes. After low-quality (>10% ambiguous bases) and mislabeled sequences were removed, a total of 3,356 sequences were aligned using MAFFT (v.7.505), and the primer outputs by PrimalScheme were mapped against this alignment in Geneious Prime (v.2023.2.1, Biomatters Ltd). The primers were manually modified (i.e., shifted upstream/downstream, application of degenerate nucleotides, or the development of alternate/supplementary primers) as required to account for any genetic diversity, correct obvious flaws within the priming region, and to ensure that the 5′ and 3′ ends of the genome were adequately captured, such that all coding regions could be recovered.

#### RSVB

The assay for RSVB was designed similarly as described above for RSVA, but with some changes to simplify the primer design process. The RSVB design was targeted against more contemporary isolates that have been isolated since 2018, which comprised a total of 2,126 partial (≥8,000 bases) and complete genomes downloaded from BVBRC (*n* = 437, accessed 2 March 2023) and GISAID (*n* = 1,692; accessed 2 March 2023). After low-quality (>10% ambiguous bases) and mislabeled sequences were removed, a total of 2,097 sequences were clustered at 99%, 98% and 97% sequence identity using cd-hit-est (v.4.8.1) (33). The set of representative sequences output by cd-hit-est from each sequence identity level was aligned using MAFFT (v.7.505) (34), and a consensus sequence was generated from each data set in Geneious Prime (v.2023.2.1, Biomatters Ltd) using the majority nucleotides at each position. The consensus sequences were used as input for PrimalScheme (v.1.3.2) with default settings, and the RSVB isolates, HRSV/B/Bern/2019 (GenBank accession no. MT107528.1), were used as the coordinate system to develop a multiplex tiling PCR scheme using default settings, except the amplicon size was set to 500 nt. The primer outputs by PrimalScheme were mapped against an alignment comprising the initial data set of 2,097 sequences and manually modified as required to account for any genetic diversity, correct obvious flaws within the priming region, and to ensure that the 5′ and 3′ ends of the genome were adequately captured such that all coding regions could be recovered.

Prior to ordering, the primers from both assays were reassessed *in silico* against their respective data sets to identify any primers that required modification (i.e., shifted up/downstream, application of degenerate nucleotides, or the development of alternate/supplementary primers) to correct for mutations in their extending ends, and/or to account for diversity not captured during the design with PrimalScheme.

### RNA extraction, quantitative real-time PCR, and cDNA synthesis

Viral RNA was extracted from 265 µL of clinical specimens using the Magmax-96 Viral RNA Isolation Kit (cat. no. AMB1836-5, Life Technologies–Invitrogen) as per the manufacturer’s protocol, and the samples were processed on the Thermo Scientific KingFisher Flex Purification System. Viral RNA was eluted in 90 µL of Tris elution buffer and either used immediately or stored at −80°C. The presence of RSV and subtypes was confirmed by quantitative real-time PCR (qRT-PCR) using Invitrogen SuperScript III Platinum One-Step qRT-PCR System (cat. no. 11732088) with RSV subtyping primers and probes based on the *L* gene (35) and listed here in Table 1. The temperature cycles were one cycle at 50°C for 30 min, one cycle at 95°C for 2 min, 45 cycles at 95°C for 15 s and 55°C for 30 s. SuperScript IV Reverse Transcriptase (cat. no. 18090200, Invitrogen) was used to synthesize cDNA from 5 µL of RNA in conjunction with 0.5 µL of 60 µM random hexamers (cat. no. S1330S, New England Biolabs [NEB]) in a final reaction volume of 10 µL. The reverse transcription reaction was incubated at 42°C for 50 min followed by 70°C for 10 min.

**TABLE 1:** Primer/probe sequences based on *L* gene used for identifying RSV subtypes

Primer/probe names	Sequences (5′–3′)
RSV_Forward	AATACAGCCAAATCTAACCAACTTTACA
RSV_Reverse	GCCAAGGAAGCATGCAATAAA
RSVA_probe	FAM-TGCTATTGTGCACTAAAG-MGBNFQ
RSVB_probe	FAM-CACTATTCCTTACTAAAGATGTC-MGBNFQ

### Preparation of primer pools and multiplex tiling PCR amplification

For each assay, multiplex primer pools were prepared for each set of primers (i.e., pool 1 and pool 2) by combining equal volumes of the appropriate primers (LabReady, 100 μM IDTE, pH 8.0; IDT) and then diluting them to 5 µM prior to use. Two separate 25 µL PCR mixtures, one for each primer pool, were prepared using the Q5 Hot Start High-Fidelity 2× Master Mix (cat. no. M0494L, NEB) as follows: 12.5 µL of Q5 2× Master Mix, 7.5 µL of nuclease-free water, 2 µL of cDNA, and 3 µL of either 5 µM primer pool 1 or pool 2. PCR amplification was carried out on a MiniAmp Plus Thermal Cycler (Applied Biosystems by Thermo Fisher Scientific) with an initial denaturation stage at 98°C for 30 s, followed by 34 cycles at 98°C for 15 s, 62°C for 5 min, and a final hold at 4°C. The PCR products from each reaction were combined then purified using an equal volume of PCRClean DX (cat. no. C-1003-250, Aline Biosciences) as per the manufacturer’s protocol. The 1× dsDNA High Sensitivity Kit (cat. no. Q33230, Invitrogen) was used to quantify 1 µL of the purified PCR product on the Qubit Flex fluorometer (Invitrogen) as per the manufacturer’s protocol, and the purified PCR products were normalized to 16 ng/µL with 0.01 M Tris in preparation for sequencing.

Each assay was then optimized iteratively against specimens available in-house to identify and correct poorly amplifying or dropout regions either by modulation of the relative primer concentrations or development of additional replacement primers. The finalized primer sequences and corresponding ratios for both assays are listed in Tables 2 and 3.

**TABLE 2:** RSVA multiplex PCR primer pools

Pool	Name	Sequences	Ratio
Pool 1	hRSVA_99_800-900_v3_1_LEFT	TGTTATTACAAGTAGTGATATTTGCCCY	5×
	hRSVA_99_800-900_v3_1_RIGHT	TTGCCCCATCTTTCATCTTATRT	5×
	hRSVA_99_800-900_v3_3_LEFT	TGAAATGAAACGTTATAAAGGYTTAYTACC	5×
	hRSVA_99_800-900_v3_3_RIGHT	AATTGGGCTTGTTCCCTRCAGT	5×
	hRSVA_99_800-900_v3_5_LEFT	GAAGCTATGGCAAGACTCAGGA	1×
	hRSVA_99_800-900_v3_5_RIGHT	TCAAGTGTGTTCAGATCTTTATTTCTGA	1×
	hRSVA_99_800-900_v3_7_LEFT	AAGCAAATTCTGGCCTTACTTTAC	1×
	hRSVA_99_800-900_v3_7_RIGHT	GTTGGATTGTTGCTGCATATGCT	1×
	hRSVA_99_800-900_v3_9_LEFT	CAAACCTCAAACCACAAAACCAAAR	2×
	hRSVA_99_800-900_v3_9_RIGHT	GCRATTGCAGATCCAACACCTA	2×
	hRSVA_99_800-900_v3_11_LEFT	TGTAACTACACCTGTAAGCACTTATATGT	1×
	hRSVA_99_800-900_v3_11_RIGHT	GTAGTTATCATGATATTTGTGGTGGATTTAC	1×
	hRSVA_99_800-900_v3_13_LEFT	ATAAGTGGAGCTGCAGAGTTGG	1×
	hRSVA_99_800-900_v3_13_RIGHT	AGGACCATTGAATATGTAACTTCCTAARG	1×
	hRSVA_99_800-900_v3_15_LEFT	CTATGCTATATTGAATAAACTGGGGCT	3×
	hRSVA_99_800-900_v3_15_RIGHT	AGGTTRTTGTCACCTGCAAGYT	3×
	hRSVA_99_800-900_v3_17_LEFT	AGATGGTTAACTTACTATAAACTAAACAC	2×
	hRSVA_99_800-900_v3_17_RIGHT	TCCATAGTTTTTGACACCACCCTT	2×
	hRSVA_99_800-900_v3_19_LEFT	AGTATAAAGAAAGTCCTAAGAGTGGGAC	1×
	hRSVA_99_800-900_v3_19_LEFT_alt1	TAGTATAAAGAAAGTCCTAAGAGTRGG	1×
	hRSVA_99_800-900_v3_19_RIGHT	GCAGAAGTCTTTTCCAGTATGTTAGT	1×
	hRSVA_99_800-900_v3_21_LEFT	ACTATAGCTAGTGGCATAATCATAGARAA	2×
	hRSVA_99_800-900_v3_21_RIGHT	TCCCTCTCCCCAATCTTTTTCAA	2×
	hRSVA_99_800-900_v3_23_LEFT	AATTACAACAAATTATATCATCCYACACC	3×
	hRSVA_99_800-900_v3_23_RIGHT	CATCTTGAGCATGATATTTTACTATTAAYGTAC	3×
	hRSVA_99_800-900_v3_25_LEFT	AGAGTGTTGTTAGTGGAGATATACTATC	5×
	hRSVA_99_800-900_v3_25_RIGHT	ACGAGAAAAAAAGTGTCAAAAACTAATRTC	5×
	hRSVA_99_800-900_v3_25_RIGHT_alt2	AATATACATATAAACCAATTAGATTTGGATTTAA	5×
Pool 2	hRSVA_99_800-900_v3_0_LEFT	CGAAAAAATGCGTACAACAAACTT	2×
	hRSVA_99_800-900_v3_0_LEFT_alt1	GGGCAAATAAGAATTTGATAAGTACCA	2×
	hRSVA_99_800-900_v3_0_RIGHT	ACTTTGTGCAATAGTTTCATTTCATAGTT	2×
	hRSVA_99_800-900_v3_2_LEFT	CCCATAATATACAAGTATGATCTCAATCCRT	1×
	hRSVA_99_800-900_v3_2_RIGHT	CCACCTCTGGTAGAAGATTGTGC	1×
	hRSVA_99_800-900_v3_4_LEFT	TGACAGCAGAAGAACTAGAGGC	1×
	hRSVA_99_800-900_v3_4_RIGHT	TCAGAAATCTTCAAGTGATAGATCATTRTC	1×
	hRSVA_99_800-900_v3_6_LEFT	GATCTCACTATGAAAACACTCAAYCC	1×
	hRSVA_99_800-900_v3_6_RIGHT	TTGACTCGAGCTCTTGGTARY	1×
	hRSVA_99_800-900_v3_8_LEFT	CCAGATCAAGAACACAACCCCAA	1×
	hRSVA_99_800-900_v3_8_RIGHT	GCAACTCCATTGTTATTTGCCCC	1×
	hRSVA_99_800-900_v3_10_LEFT	CAAAGCACACCAGCAGCMAA	2×
	hRSVA_99_800-900_v3_10_RIGHT	TTGAATATGTCAATGTTGCAGAGATTTAC	2×
	hRSVA_99_800-900_v3_12_LEFT	TCCCYTCTGATGAATTTGATGCA	1×
	hRSVA_99_800-900_v3_12_RIGHT	TGAGTTCAGTRAGGAGTTTGCTCA	1×
	hRSVA_99_800-900_v3_14_LEFT	TGATACTACCTGACAAATATCCTTGTAG	1×
	hRSVA_99_800-900_v3_14_RIGHT	TTGTTTTGWGGATTGATTTTTGYCTG	1×
	hRSVA_99_800-900_v3_14_RIGHT_alt1	TATTAACCATGATGGAGGATGTTGC	1×
	hRSVA_99_800-900_v3_16_LEFT	TGCTCAACAACATCACAGATGC	1×
	hRSVA_99_800-900_v3_16_RIGHT	TCTGCTAATATTTGAACTTGTCTGAACA	1×
	hRSVA_99_800-900_v3_18_LEFT	TCCTGGTTACATTTAACTATTCCTCATG	1×
	hRSVA_99_800-900_v3_18_RIGHT	AGGAAATCAGGAGTTCTTCTATAGAAACT	1×
	hRSVA_99_800-900_v3_20_LEFT	AACCTACATATCCTCAYGGGCT	2×
	hRSVA_99_800-900_v3_20_RIGHT	AGCTAAGGCCAAARCTTATACAGT	2×
	hRSVA_99_800-900_v3_22_LEFT	TCTMATGTTAATTCTAATTTAATATTGGCRCA	5×
	hRSVA_99_800-900_v3_22_RIGHT	TCTTGTYTGCTGTAATTGGTTCTAATC	5×
	hRSVA_99_800-900_v3_24_LEFT	GTTCTACAGGTTGTAAAATTAGTATAGAGT	3×
	hRSVA_99_800-900_v3_24_RIGHT	CTGTTGATCTGAAATTTAAAACATGRTTGAACC	3×

**TABLE 3:** RSVB multiplex PCR primer pools

Pool	Name	Sequences	Ratio
Pool 1	hRSVB_2018-2023_500_1_LEFT	CTAGACTCCGTCACGCGAAAAA	2×
	hRSVB_2018-2023_500_1_LEFT_alt1	AAATGCGTACTACAAACTTGCACACTC	2×
	hRSVB_2018-2023_500_1_RIGHT	GGAGATCAAGCCCAAGTAAATCAGA	2×
	hRSVB_2018-2023_500_3_LEFT	TCAATTATGAGATGAAGCTATTGCACA	1×
	hRSVB_2018-2023_500_3_RIGHT	TGCATCTTCAGTGATTAATAGCATACC	1×
	hRSVB_2018-2023_500_5_LEFT_alt1	CCAGACTGTGGGATGATAATACTGTG	1×
	hRSVB_2018-2023_500_5_RIGHT	TTGCCTAGGACCACACTTGAGA	1×
	hRSVB_2018-2023_500_7_LEFT_alt1	CAAGTTTGCATCATCCAAAGATCCTAA	1×
	hRSVB_2018-2023_500_7_RIGHT_alt1	TCTTGCCATGGCCTCTAACCTAT	1×
	hRSVB_2018-2023_500_7_RIGHT_alt2	GCCTCTAACCTATCATTGGTCAT	1×
	hRSVB_2018-2023_500_9_LEFT	GGAAACATACGTGAACAAGCTTCA	1×
	hRSVB_2018-2023_500_9_RIGHT_alt1	ACACTGATTGATCTTAGATAGGTTGGTATT	1×
	hRSVB_2018-2023_500_11_LEFT_alt1	TCCTCAACTGCACACTATATCTAAACATC	1×
	hRSVB_2018-2023_500_11_RIGHT	TGTGATGCCATGACTCTGTGAG	1×
	hRSVB_2018-2023_500_13_LEFT	CACAAAGTTACACTAACAACTGTCACA	10×
	hRSVB_2018-2023_500_13_RIGHT_alt1	TCTGTAGTCTTGGGGGTTGGTTT	10×
	hRSVB_2018-2023_500_15_LEFT_alt1	CTGGGGCAAATAACCATGGAGY	1×
	hRSVB_2018-2023_500_15_RIGHT_alt1	TGTTCACTTCTCCTTCAAGGTG	1×
	hRSVB_2018-2023_500_17_LEFT	TCAATGATATGCCTATAACAAATGATCAGA	5×
	hRSVB_2018-2023_500_17_RIGHT_alt1	TCCACGATTTTTGTTGGATGC	5×
	hRSVB_2018-2023_500_19_LEFT	TCGTAGATCCGATGAATTATTACATAATGT	1×
	hRSVB_2018-2023_500_19_RIGHT	TGACTGTAGTGGCATCTTCTACC	1×
	hRSVB_2018-2023_500_21_LEFT	CAATACTGTTATATCATACATCGAGAGCA	1×
	hRSVB_2018-2023_500_21_RIGHT_alt1	GGATCCATTTTGTCCCATAACTTTATTRAG	1×
	hRSVB_2018-2023_500_23_LEFT	ATAAAAGCATGTCCTCGTCTGAAC	2×
	hRSVB_2018-2023_500_23_RIGHT	AGCCCTTTATGATAAACAATGCAACC	2×
	hRSVB_2018-2023_500_25_LEFT_alt1	TCACAGATGCAGCTATTAAGGC	1×
	hRSVB_2018-2023_500_25_RIGHT	ACTCACGATAGAACCGCAATCC	1×
	hRSVB_2018-2023_500_27_LEFT	TGCAACCAGGTATGTTTAGGCAA	1×
	hRSVB_2018-2023_500_27_RIGHT	AATGATATGGCTTCAATGGTCCAC	1×
	hRSVB_2018-2023_500_29_LEFT_alt1	AGGTCCATGGATAAATACAATACTTGATGA	2×
	hRSVB_2018-2023_500_29_RIGHT	GCCTGTGGATCCCTCATCAATG	2×
	hRSVB_2018-2023_500_31_LEFT	AAAAACATCAGCGATAGATACAACTGA	1×
	hRSVB_2018-2023_500_31_RIGHT_alt1	ATGACAGTCCAAGTGTTCCAGTAC	1×
	hRSVB_2018-2023_500_31_RIGHT_alt2	CATATGACAGTCCAAGTGTTCC	1×
	hRSVB_2018-2023_500_33_LEFT	ACATTTGATGAAACCTCCTATATTTACAGG	2×
	hRSVB_2018-2023_500_33_RIGHT	TGACTTTTTGTTCTAGGAAAACTTTAGACA	2×
	hRSVB_2018-2023_500_35_LEFT_alt1	CTGCTAACAAAACAAATAAGGATTGCTAA	1×
	hRSVB_2018-2023_500_35_RIGHT_alt1	ACCAGCTCCTTCACCTATGAATG	1×
	hRSVB_2018-2023_500_37_LEFT	TGGAGTAAGCATGTAAGAAAGTGCA	1×
	hRSVB_2018-2023_500_37_RIGHT_alt1	TCTAAAGTTTAAAACATGATCCAGCCAT	1×
Pool 2	hRSVB_2018-2023_500_2_LEFT_alt1	TGCTCTCAATTAAATGGTCTAATAGATGATAA	1×
	hRSVB_2018-2023_500_2_RIGHT_alt1	GCATAGGGAATGTGCCATATTTTGTA	1×
	hRSVB_2018-2023_500_4_LEFT_alt1	ACACTATTCAACGTAGTACAGGAGA	1×
	hRSVB_2018-2023_500_4_RIGHT	CATTGTTTGCCCTCCTAATTACTGC	1×
	hRSVB_2018-2023_500_6_LEFT_alt1	CAGAAGTTGGGAGGAGAAGC	1×
	hRSVB_2018-2023_500_6_RIGHT	TGTTGGTGCCAGATGTTATCGG	1×
	hRSVB_2018-2023_500_8_LEFT_alt1	CTCGTGACGGAATAAGAGATGC	1×
	hRSVB_2018-2023_500_8_RIGHT	TGATGCGGGATCATCATCTTTTTC	1×
	hRSVB_2018-2023_500_10_LEFT	ACCCCACTCATGAGATCATTGC	1×
	hRSVB_2018-2023_500_10_RIGHT	GCAGACAATGGCTGGAAGTGAT	1×
	hRSVB_2018-2023_500_12_LEFT_alt1	TCGACACATAGTGTTCTCCCATTAT	10×
	hRSVB_2018-2023_500_12_RIGHT_alt1	GGCTAACCCTTTCTGGTGAGACT	10×
	hRSVB_2018-2023_500_14_LEFT_alt1	ACCACAAACAAAAGAGACYCYA	10×
	hRSVB_2018-2023_500_14_RIGHT_alt1	CCTCAGTTATGTTCTGACTTGAGGY	5×
	hRSVB_2018-2023_500_16_LEFT_alt1	AGGAAACGAAGATTTCTGGGCTT	1×
	hRSVB_2018-2023_500_16_RIGHT_alt1	AGCTGTACAACATATGCAAGGAC	1×
	hRSVB_2018-2023_500_18_LEFT_alt1	CAAAAACAGACATAAGCAGCTCAG	1×
	hRSVB_2018-2023_500_18_RIGHT	TGATTCCACTTAGTTGGTCTTTGCT	1×
	hRSVB_2018-2023_500_20_LEFT	TCACAAAACTAACAGCTGGGGC	1×
	hRSVB_2018-2023_500_20_RIGHT	TTGTCTTCTTCAGCACGTCTGC	1×
	hRSVB_2018-2023_500_22_LEFT	ACATCTTAACATCCCTGAAGATATATATACAGT	2×
	hRSVB_2018-2023_500_22_RIGHT_alt1	AGTTTATTCAAGATGGCGTACACYT	2×
	hRSVB_2018-2023_500_24_LEFT_alt1	TCAAATGAGGTAAAAAGTCATGGGT	2×
	hRSVB_2018-2023_500_24_RIGHT	ACCATTTATGATATTATCAGACACTGTCTT	2×
	hRSVB_2018-2023_500_26_LEFT	GCTATTGTCCTACCTCTAAGATGGT	1×
	hRSVB_2018-2023_500_26_RIGHT_alt1	TGTCAAACTCTCAGGGAAGAATTG	1×
	hRSVB_2018-2023_500_28_LEFT_alt1	TGAAGTTGATGAACAAAGTGGGTTA	2×
	hRSVB_2018-2023_500_28_RIGHT_alt1	ACTGCATAATAAGCTTTCTCCTCTGTA	2×
	hRSVB_2018-2023_500_30_LEFT_alt1	AGCTCCAGGATCTTCCAGAYGAT	2×
	hRSVB_2018-2023_500_30_RIGHT	TCTCTTTTGTCTTTGTTACAATCTAGTGG	2×
	hRSVB_2018-2023_500_32_LEFT_alt1	CCAAATAGATTTATTAGCAAAATTAGACTGG	2×
	hRSVB_2018-2023_500_32_RIGHT_alt1	TTGGGTTAAACTTATTTTATCTGGTAGGAAC	2×
	hRSVB_2018-2023_500_34_LEFT_alt1	GCAAAATTAGAATGTGATATGAACACTTCAGA	2×
	hRSVB_2018-2023_500_34_RIGHT	ACAGGGATTAATGATACATTTTCTAAAGCT	2×
	hRSVB_2018-2023_500_36_LEFT_alt1	CCTTGGCATCATGTCAATAGATTTAACTT	1×
	hRSVB_2018-2023_500_36_RIGHT	TGAAATCAATATCATCTTGAGCATGGT	1×
	hRSVB_2018-2023_500_38_LEFT	ACTTCATTGTCAAAATTGAAGAGTGTAGT	1×
	hRSVB_2018-2023_500_38_RIGHT	CATGCCGGCCACGAGAAAAA	2×
	hRSVB_2018-2023_500_38_RIGHT_alt1	AATGTCTCGTTGTGTTGTAAATGCACATR	2×

### Nanopore library preparation and sequencing workflow

A total of 12.5 µL of normalized PCR product (~200 ng) from each sample was used as input to generate barcoded sequencing libraries using the Native Barcoding Kit (cat. no. SQK-NBD114-96, ONT). The protocol was followed exactly with the exception that the NEBNext FFPE DNA Buffer and Repair Mix were not used and replaced with 1.75 µL of Ultra II End-prep Reaction Buffer. The final library, comprising 12 µL of the pooled libraries, 37.5 µL of sequencing buffer, and 25.5 µL of library beads, was loaded onto a FLO-MIN114 R10.4.1 flow cell with a max of 96 specimens and sequenced for 72 h on the MinION Mk1C Sequencing Platform.

### Bioinformatics analysis of sequence data obtained from Nanopore platform

The FAST5 files were basecalled and demultiplexed using Guppy (v.6.5.7) with default settings except specification of dna_r10.4.1_e8.2_400bps_5khz_hac as the basecaller model and SQK-NBD114-96 as the barcode kit with the “--require_barcode_both_ends” parameter. The basecalled FASTQ files were processed using the Nextflow-enabled viralassembly pipeline (https://github.com/phac-nml/viralassembly), which is a genericized version of the ncov2019-artic-nf pipeline (https://github.com/connor-lab/ncov2019-artic-nf) that automates the ARTIC Network’s Field Bioinformatics Toolkit (https://github.com/artic-network/fieldbioinformatics). The viralassembly pipeline is capable of processing any multiplex tiling PCR assay as long as the reference sequence (fasta format) and primer coordinate file (bed format) used to create the assay are provided (File S1 through S6). Briefly, the viralassembly pipeline automates read mapping, primer trimming, variant calling, and consensus sequence generation. Default settings were used, except Medaka (https://github.com/nanoporetech/medaka) was selected as the variant caller in conjunction with the r1041_e82_400bps_hac_v4.2.0 model, and the maximum read length to keep was set to 1,500 nt for the RSVA assay and 1,000 nt for the RSVB assay.

For the purposes of this study, a genome was considered to be complete if it encompassed the first codon of the NS1 protein and the last codon of the L protein (15). The open reading frames (ORFs) encoding the RSV genes were identified using Geneious Prime (v.2023.2.1, Biomatters Ltd) and characterized as a means to detect and correct any sequencing errors or to validate legitimate biological mutations that would result in the formation of a truncated protein.

### RSVA and RSVB data sets

In order to contextualize our sequences within the broader population structure of RSV, 124 RSVA and 83 RSVB lineage exemplar reference sequences (https://github.com/rsv-lineages) were downloaded from the National Center for Biotechnology Information (NCBI). They were combined with the 52 RSVA and 40 RSVB sequences generated here (37 Canadian + 3 American Type Culture Collection [ATCC] isolates), resulting in data sets comprising 176 RSVA and 123 RSVB sequences, respectively (File S7).

### Characterization of RSVA and RSVB sequences using Nextclade

The RSVA and RSVB data sets were processed using Nextclade’s web portal on 9 August 2024 (https://clades.nextstrain.org/) and analyzed using the RSVA module against the reference isolate hRSV/A/England/397/2017 (EPI_ISL_412866) and the RSVB module against the reference isolate hRSV/B/Australia/VIC-RCH056/2019 (EPI_ISL_1653999), respectively. The RGCC lineage assignments and G_Clade typing nomenclatures (15, 36), as well as quality metrics and genomic features including the genome length, breadth of coverage, and the presence of variants including nt and aa substitutions, insertions, deletions and frameshifts, are described in File S7. Amino acid changes identified in the F protein among the 52 RSVA and 37 RSVB sequences were tabulated and used to generate heatmaps depicting their carriage across the data sets. The heatmaps were generated using a custom R script implemented in RStudio 2023.12.1 Build 402 running R (v.4.3.2, 31 October 2023) with the following packages: ComplexHeatmap (v.2.10.0), tidyverse (v.2.0.0), magrittr (v.2.0.3), grid (v.4.1.2), and vegan (v.2.6.4). The R code used to generate the heatmaps is contained in File S8.

### Whole genome phylogenetic analysis

The RSVA and RSVB data sets were aligned separately with MAFFT (v.7.520) using default settings (34), then processed with FastTree to infer approximate maximum-likelihood phylogenetic trees with bootstrapping using default settings and the generalized time reversible (GTR) model (37, 38). The phylogenetic trees were visualized using the Interactive Tree Of Life (iTOL) tool (39) and annotated with the corresponding sequence metadata captured in File S7.

### *Glycoprotein* (*G*) gene phylogenetic analysis

The G ORF for all RSVA and RSVB samples was identified using the “Find ORFs” function in Geneious Prime (v.2023.2.1, Biomatters Ltd), and the corresponding coding sequences were extracted and subjected to BLAST analysis to confirm they were in-frame and intact. The G sequences from RSVA and RSVB were each aligned separately using MAFFT (v.7.520) with default settings (34), then maximum-likelihood trees were inferred using FastTree with default settings and the GTR model (37, 38). The phylogenetic trees were visualized using the iTOL tool (39) and annotated with the corresponding RGCC lineage and G_Clade assignments from Nextclade, as well as the sample collection year and location (File S7).

### Co-phylogeny analysis

For both RSVA and RSVB, a custom R script was used to construct a co-phylogeny comprising the tree files generated from the whole genome and *G* gene coding sequences, and annotated with the corresponding RGCC lineage for each sample. The script was implemented as described above using the following packages managed with pacman (v.0.5.1): ape (v.5.7.1), biocmanager (v.1.30.22), tidyverse (v.2.0.0), ggtree (v.3.10.1), phangorn (v.2.11.1), treeio (v.1.26.0), phytools (v.2.1.1), viridis, here (v.1.0.1), and scico (v.1.5.0). The R code used to generate the co-phylogeny tree is contained in File S8.

### Identification of mutations linked to phenotypic or epidemiological traits using RSVsurver

The RSVsurver, developed by Singapore’s Agency for Science, Technology and Research Bioinformatics Institute and enabled by GISAID (https://rsvsurver.bii.a-star.edu.sg/faq.html), was used to screen the RSV sequences against their curated database for the presence of mutations that may be linked to important phenotypic and epidemiological traits. The RSVA and RSVB data sets were uploaded to the RSVsurver, and each sequence was compared against the reference isolates hRSV/A/England/397/2017 and hRSV/B/Australia/VIC-RCH056/2019, respectively.

## RESULTS

### Multiplex PCR-based amplification using tiling scheme of RSV and complete genome sequences collection

For RSVA, PrimalScheme generated an assay comprising 24 overlapping amplicons with 0 gaps accounting for 95.5% of the genome. Additional primer sets were manually designed to generate amplicons capturing regions closer to the 5′ and 3′ ends of the genome extending coverage of the assay to >99% of the genome. For RSVB, PrimalScheme generated an assay comprising 38 overlapping regions with 0 gaps accounting for 99.6% of the genome. The finalized primer sequences and corresponding ratios for both assays are listed in Tables 2 and 3. Among the in-house 52 RSVA and 37 RSVB clinical specimens, 3 ATCC RSVB stains, and Nextclade curated NCBI 124 RSVA and 83 RSVB reference genomes, the antigenic group, subtypes, genome length, identified mutations, and GISAID IDs are summarized in File S7. The near-complete genome length ranged from 14,994 to 15,225 nt.

### Whole genome phylogenetic analysis of 52 Canadian clinical RSVA isolates

**Fig 1:**
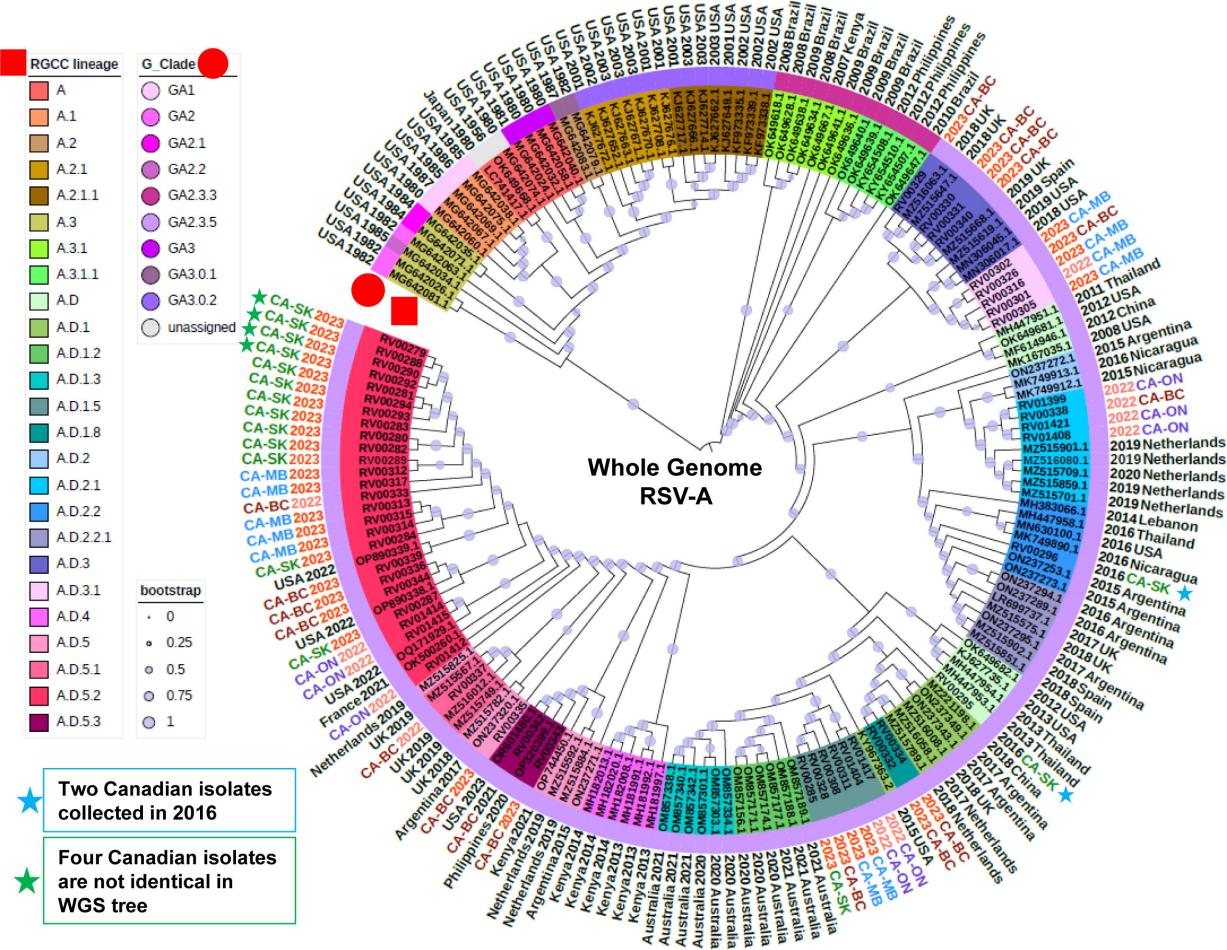
A phylogenetic tree comprising the 176 RSVA genomes used in this study, including 52 sequences derived from Canadian isolates and 124 Nextclade reference sequences. hRSV Genotyping Consensus Consortium (RGCC) lineages are indicated as colored highlights over the isolate identifier and further annotated with a red square, while the G_Clade classifications are indicated as a color strip above the isolate identifier and further annotated with a red circle. Collection years 2022 and 2023 are indicated with light red and red texts, respectively, while all other collection years are indicated with black text. The Canadian isolates were primarily collected between 2022 and 2023 from four provinces, including British Columbia (BC), Manitoba (MB), Ontario (ON), and Saskatchewan (SK), with corresponding text colors red, blue, purple, and green, respectively. Bootstrap values are annotated as light purple dots on each branch of the tree.

The RSVA data set (*n* = 176; 52 in-house + 124 reference) was demarcated into nine clades using the G_Clade scheme developed by Goya et al. (36) (File S7; Fig. 1). The majority of sequences were classified as GA.2.3.5 and largely represent more contemporaneous isolates collected since 2007, including all Canadian isolates sequenced in this study (Fig. 1). In contrast, the new scheme proposed by the hRSV RGCC and implemented in Nextclade, was more discriminatory and demarcated the same data set into 25 lineages that more closely reflect the structure of the phylogenetic tree (Fig. 1, represented by colored range with the tree nodes). Overall, lineage assignment was well supported by the phylogenetic tree, though not all sequences from lineages A.D and A.D.5 clustered cohesively. However, this was also apparent in the RSVA maximum-likelihood tree described in Goyal et al. (15) and the publicly available Nextclade RSVA phylogeny.

Isolates derived from clinical specimens characterized in this study, collected between 2016 and 2023, were assigned to nine lineages, all descending from A.D, with the plurality of sequences (25 out of 52) assigned to lineage A.D.5.2 (Fig. 1). The two oldest isolates, collected from SK in 2016, were assigned to lineages A.D and A.D.2.2, respectively, and clustered with reference sequences collected between 2012 and 2016 (blue stars, Fig. 1). Based on available data, lineage A.D.2.2 was prevalent among isolates characterized in 2016, but detections have declined since then (15). Interestingly, the remaining Canadian isolates, collected during the 2022–2023 respiratory virus season and one from 2021, were assigned to multiple lineages, indicating that a diverse array of viruses can be co-circulating simultaneously. With the exception of A.D.5.2 and A.D.1, which represent the most common (*n* = 25) and second most common (*n* = 8) lineages detected among the Canadian isolates, a lineage tended to be dominated by sequences from a single province (excluding reference sequences) (Fig. 1).

### RSVA *G* gene-based phylogenetic analyses and its co-phylogeny analysis with whole genome-based tree

**Fig 2:**
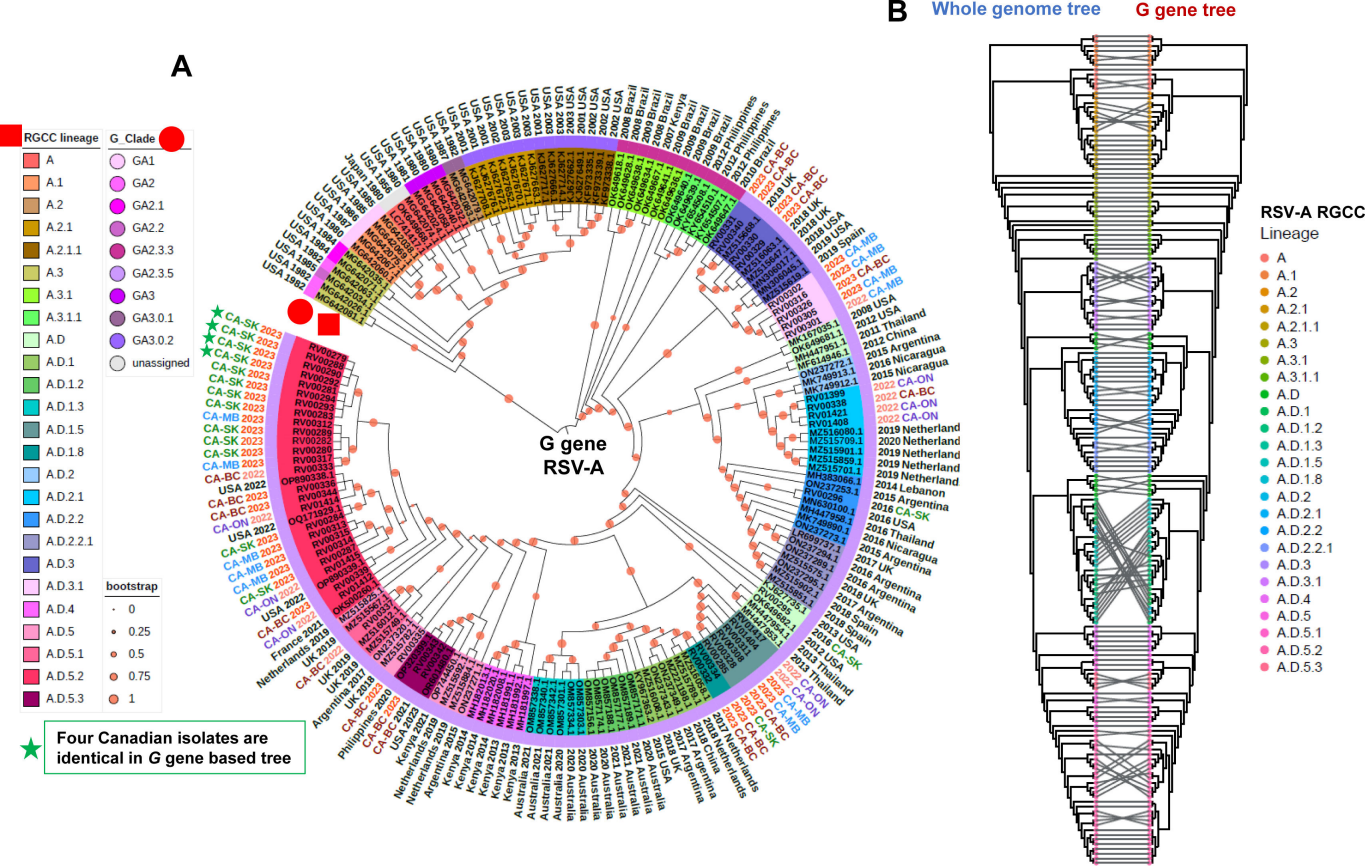
A phylogenetic tree derived from the *G* gene sequence and a co-phylogeny comparing differences in clustering using the whole genome sequence versus only the corresponding *G* gene sequence based on the 176 RSVA genomes used in this study. (**A**) A phylogenetic tree based on the *G* gene sequences. The RSV RGCC lineages are indicated as colored highlights over the isolate identifier and further annotated with a red square, while the G_Clade classifications are indicated as a color strip above the isolate identifier and further annotated with a red circle. Collection years 2022 and 2023 are indicated with light red and red texts, respectively, while all other collection years are indicated with black text. The Canadian isolates were primarily collected between 2022 and 2023 from four provinces, including BC, MB, ON, and SK, with corresponding text colors red, blue, purple, and green, respectively. Bootstrap values are annotated as red dots on each branch of the tree. (**B**) A co-phylogeny comparing differences in clustering using the whole genome versus only the corresponding *G* gene sequence. The corresponding RGCC lineage is annotated at each tip for both phylogenetic trees.

The structure of the phylogenetic tree derived from the complete coding sequence of the *G* gene (Fig. 2A) was largely congruent with the phylogeny derived from the whole genome sequence (Fig. 1). Overall, the lineage designations corresponded to well-defined groups in both phylogenies, though the use of the whole genome sequence showed higher bootstrap values and was better able to resolve the phylogenetic structure within certain sublineages, such as A.D.5.2, and differentiated closely related sequences that were indistinguishable based on the *glycoprotein G* gene sequence (Fig. 1 and 2A). For instance, within the A.D.5.2 lineage, four isolates (RV00279 [CA-SK 2023], RV00288 [CA-SK 2023], RV00290 [CA-SK 2023], and RV00292 [CA-SK 2023]) (green stars, Fig. 2A) were indistinguishable using the *G* gene sequence, but at the whole genome sequence level, could be resolved into two pairs of identical sequences: RV00279 (CA-SK 2023) and RV00288 (CA-SK 2023), as well as RV00290 (CA-SK 2023) and RV00292 (CA-SK 2023) (green stars, Fig. 1).

### Whole genome phylogenetic analysis of 37 Canadian clinical RSVB isolates

**Fig 3:**
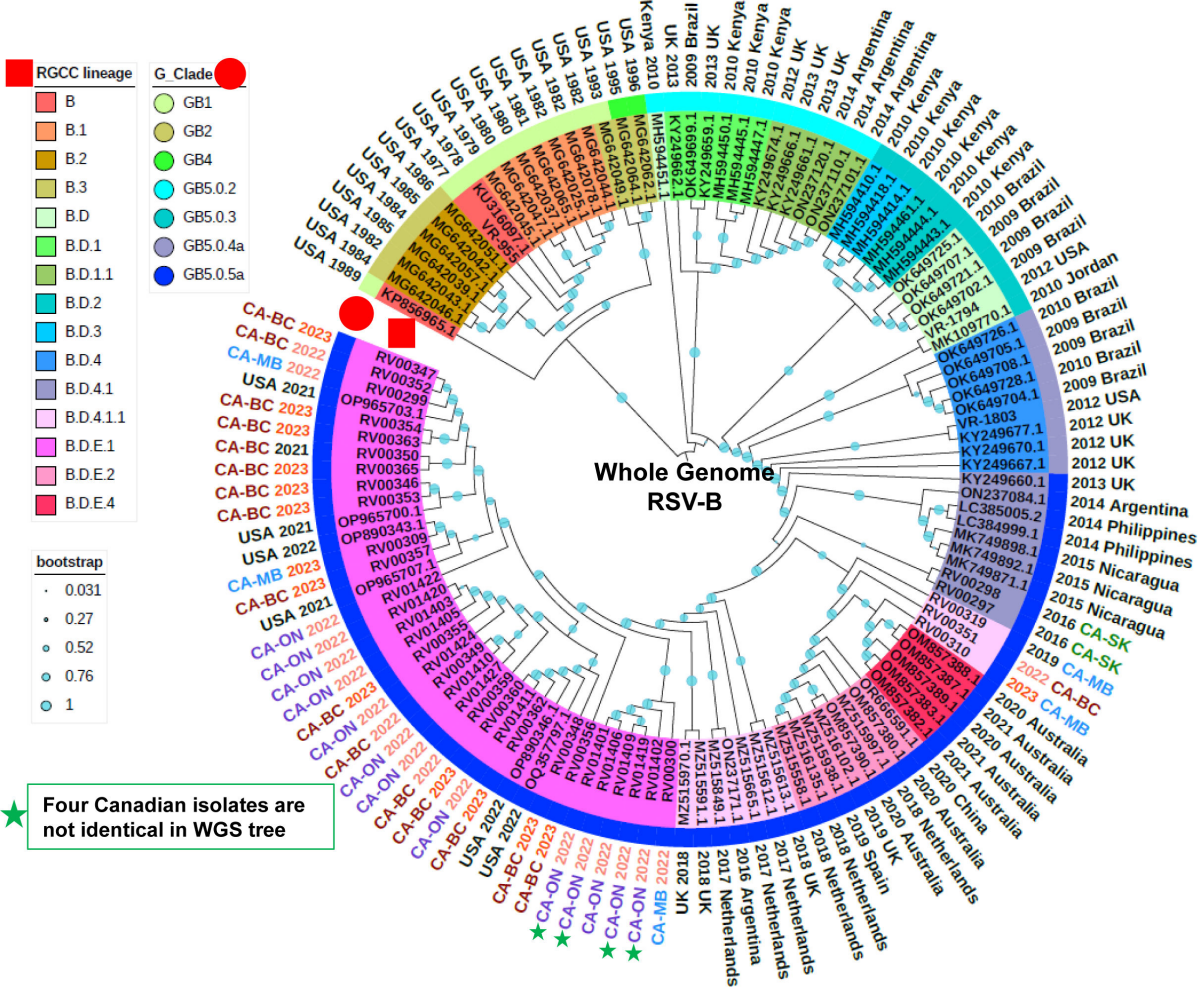
A phylogenetic tree comprising the 123 RSVB genomes used in this study, including 37 sequences derived from Canadian isolates, 3 ATCC isolates, and 86 Nextclade reference sequences. The RSV RGCC lineages are indicated as colored highlights over the isolate identifier and further annotated with a red square, while the G_Clade classifications are indicated as a color strip above the isolate identifier and further annotated with a red circle. Collection years 2022 and 2023 are indicated with light red and red text, respectively, while all other collection years are indicated with black text. The Canadian isolates were primarily collected between 2022 and 2023 from four provinces, including BC, MB, ON, and SK, with corresponding text colors red, blue, purple, and green, respectively. Bootstrap values are annotated as cyan dots on each branch of the tree.

Using the new RSVB scheme developed by the RGCC, the RSVB data set (*n* = 123; 37 Canadian + 86 references) was demarcated into 15 lineages that largely reflect the structure of the phylogenetic tree (Fig. 3). Comparatively, using the G_Clade scheme developed by Goya et al. (36), the data set was characterized across seven clades with the majority of sequences classified as GB5.05a, encompassing isolates collected since 2013, including all Canadian isolates sequenced in this study (Fig. 3; File S7). One sequence from each of the B (KP856965) and B.D (MH594451) lineages did not cluster cohesively with other sequences from their respective lineages, but this is also reflected in the RSVB maximum-likelihood tree described in Goya et al. (15) and the publicly available Nextclade RSVB phylogeny. Isolates derived from the Canadian clinical specimens were demarcated into three lineages with the majority (30 out of 37) assigned to B.D.E.1, which included those from the 2022–2023 respiratory virus season from all provinces, as well as one collected in 2021 from BC. The two isolates collected in 2016 from SK were assigned to B.D.4.1 and clustered with reference sequences collected between 2013 and 2015, while the remaining three isolates, two collected from MB in 2019 and 2023 and one collected from BC in 2022, were assigned to B.D.4.1.1, and were more distantly related to their corresponding NCBI reference isolates (Fig. 3). The reference viruses VR-955 (collected in 1977), VR-1794 (collected in 2012), and VR-1803 (collected in 2012) were assigned to lineages B, B.D, and B.D.4, respectively, and clustered with appropriately contemporaneous reference sequences.

### RSVB *G* gene-based phylogenetic analyses and its co-phylogeny analysis with whole genome-based tree

**Fig 4:**
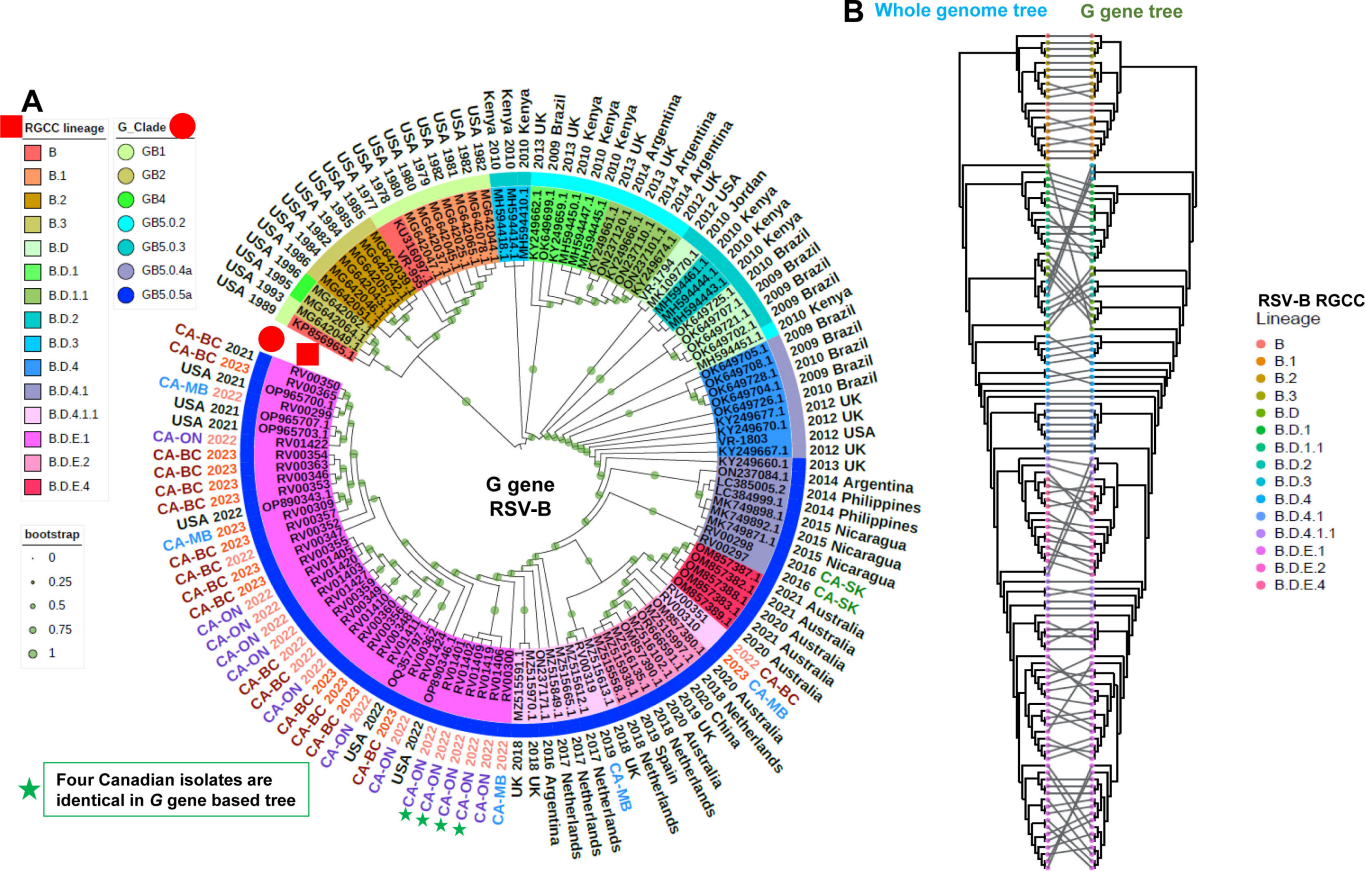
A phylogenetic tree derived from the *G* gene sequence and a co-phylogeny comparing differences in clustering using the whole genome sequence versus only the corresponding *G* gene sequence based on the 123 RSVB genomes used in this study. (**A**) A phylogenetic tree based on the *G* gene sequences. The RSV RGCC lineages are indicated as colored highlights over the isolate identifier and further annotated with a red square, while the G_Clade classifications are indicated as a color strip above the isolate identifier and further annotated with a red circle. Collection years 2022 and 2023 are indicated with light red and red texts, respectively, while all other collection years are indicated with black text. The Canadian isolates were primarily collected between 2022 and 2023 from four provinces, including BC, MB, ON, and SK, with corresponding text colors red, blue, purple, and green, respectively. Bootstrap values are annotated as light green dots on each branch of the tree. (**B**) A co-phylogeny comparing differences in clustering using the whole genome versus only the corresponding *G* gene sequence. The corresponding RGCC lineage is annotated at each tip for both phylogenetic trees.

Similar to RSVA, the structure of the phylogenetic trees derived for RSVB from the complete coding sequences of the *G* gene (Fig. 4A) and whole genome sequence (Fig. 3) was largely congruent (Fig. 4B). Overall, the lineage designations corresponded to well-defined groups in both phylogenies, though the use of the whole genome sequences was better able to resolve the phylogenetic structure within certain sublineages, such as B.D.E.1 and differentiated closely related sequences that were indistinguishable based on the *G* gene sequence (Fig. 3 and 4A). For instance, in the B.D.E.1 lineage, sequences from four isolates, including RV01401 (CA-ON 2022), RV01402 (CA-ON 2022), RV01409 (CA-ON 2022), and RV01419 (CA-ON 2022) (green stars, Fig. 4A) were indistinguishable, but at the whole genome sequence level, could be further resolved into separate clusters (green stars, Fig. 3 and 4A).

### Characterization of aa substitutions identified among the Canadian RSVA and RSVB sequences

**Fig 5:**
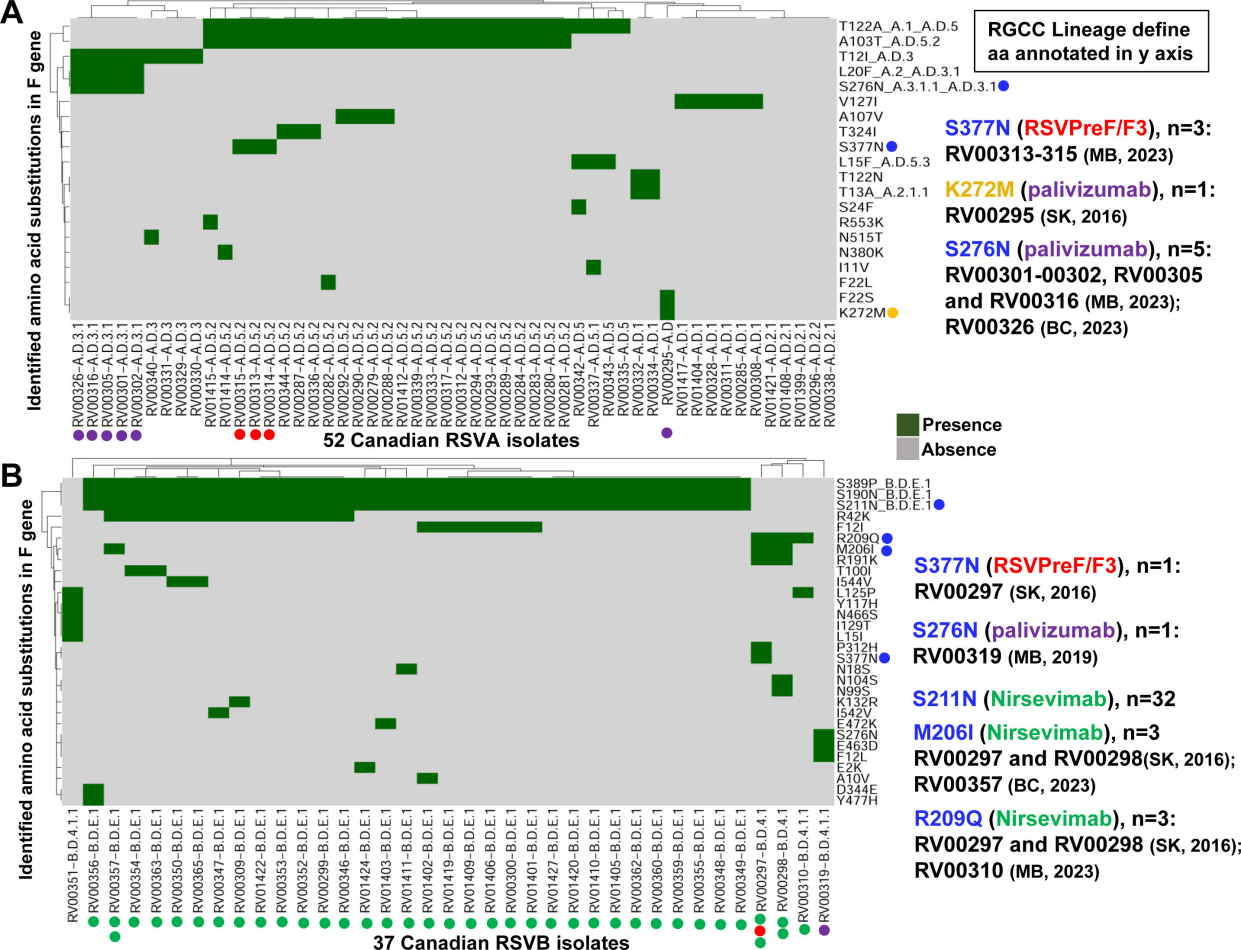
A heatmap describing the distribution of amino acid substitutions identified in the *F* gene among the (**A**) 52 Canadian RSVA and (**B**) 37 Canadian RSVB isolates. The dark green and light gray colors represent the presence or absence of a particular substitution. The isolate identifier and lineage classification corresponding to each isolate are listed on the *x*-axis, while the amino acid substitutions identified in this study are listed on the *y*-axis. Substitutions written in blue font and annotated with blue circles on the *y*-axis correspond to mutations assigned as interest level 1 (moderately significant) by RSVsurver, and similarly, those written in orange font and annotated with orange circles on the *y*-axis correspond to mutations assigned as interest level 2 (significant, known to be involved in drug binding). Isolates with substitutions in the antigenic sites corresponding to the RSVpreF/F3 vaccines and/or the mABs (palivizumab and nirsevimab) are annotated on the *x*-axis with red, purple, and green circles, respectively. Lastly, some substitutions on the *y*-axis are further annotated with RGCC lineages as they are defining features of that lineage.

Among the 52 Canadian RSVA isolates sequenced in this study, a total of 271 unique aa substitutions were observed across all proteins (NS1 = 4, NS2 = 4, N = 9, P = 11, M = 6, SH = 4, G = 100, F = 20, M2−1 = 14, M2−2 = 13, and L = 86) ranging in frequency from 1.9% to 100.0% relative to hRSV/A/England/397/2017. Notable mutations flagged by RSVsurver include F:T122A (*n* = 29) and F:T122N (*n* = 2), which negate potential glycosylation of residue 120 (magenta mutations, ), as well as F:K272M (*n* = 1; RV00295, CA-SK 2016, A.D) and F:S276N (*n* = 5; RV00301-00302, RV00305, RV00316, and RV00326) (Fig. 5A). Likewise, for the 37 Canadian RSVB isolates sequenced here, a total of 228 unique aa substitutions were observed across all proteins (NS1 = 3, NS2 = 8, N = 8, P = 6, M = 22, SH = 4, G = 62, F = 30, M2−1 = 4, M2−2 = 10, and L = 71) ranging in frequency from 2.7% to 100.0% relative to hRSV/B/Australia/VIC-RCH056/2019. RSVsurver flagged the presence of F:R191K in two isolates, RV00297 and RV00298 (CA-SK 2016, B.D.4.1) (orange mutation, ), which was shown to be among residues capable of modulating RSV fusion activity *in vitro* (40).

For the Canadian RSVA isolates, no variability was observed within the aa residues corresponding to the nirsevimab targeted antigenic site Ø (62–96 and 195–227), while variability was observed at two residues corresponding to the palivizumab targeted antigenic site II (254–277), including F:K272M (*n* = 1), as flagged by RSVsurver, as well as F:S276N (*n* = 5). Variability was also observed in one residue, F:S377N (*n* = 3), which is within the RSVPreF3 targeted antigenic site III (45–54, 301–311, 345–352, and 367–378). For RSVB, 35 out of 37 (94.6%) isolates showed variability within the aa residues corresponding to the nirsevimab targeted antigenic site Ø including F:M206I (*n* = 3), F:R209Q (*n* = 3), and F:S211N (*n* = 32), while F:S276N (*n* = 1) represented the only variability observed within antigenic site II, and F:S377N (*n* = 1) was the only change observed within antigenic site III (Fig. 5; Fig. S1 and S2 ).

## DISCUSSION

The recent licensing of two new RSV vaccines and an additional monoclonal antibody for use in Canada and abroad necessitates implementation of a more robust surveillance system to monitor the evolution of this pathogen. To that end, we and others have recognized the utility of the multiplex tiling PCR approach and developed assays for both RSVA and RSVB that can be used to quickly generate near-complete genome sequences from clinical specimens (30–32, 41, 42). Enrichment by PCR is less expensive and complex, faster, and more portable relative to other methods, such as capture probe-based assays, or using techniques like ultracentrifugation and host RNA depletion in combination with brute-force metagenomic sequencing, which is attractive for outbreak investigations, surveillance programs, and use in clinical settings. However, the primer design and subsequent multiplex PCR optimization can be challenging, especially for more diverse viral species, and primers may need to be updated over time as the targeted virus evolves (30). With the continued democratization of next-generation sequencing and the development of more accessible computational tools for assay design (i.e., PrimalScheme), multiplex tiling PCR assays are increasingly being developed and deployed to support outbreak investigation and response (i.e., Zika), as well as for large-scale genomic epidemiology initiatives (i.e., severe acute respiratory syndrome coronavirus 2) (30, 43).

Here, we conducted a small pilot study to test our assays against RSV-positive clinical specimens collected from four different provinces in Canada between 2016 and 2023. A total of 52 RSVA and 37 RSVB near-complete genomes were recovered and subjected to downstream phylogenetic and comparative genomic analyses. To help contextualize the Canadian sequences within the overall population structure of RSV, we downloaded a collection of lineage exemplar reference sequences and generated phylogenetic trees based on the whole genome and the *G* gene coding sequences (File S7). Nextclade was used to assign both the new RGCC lineage and G_Clade designations for each sequence, and these data were overlaid onto the phylogenetic trees (Fig. 1 to 4). We observed that the overall structures of the phylogenetic trees derived from the WGS-based and G sequences were largely congruent, though the former was able to distinguish more closely related isolates, given the presence of additional sequence data available for interrogation (Fig. 1, 2A to 4A). This highlights the sensitivity and utility of the whole genome approach to support high-resolution outbreak and trace-back investigations. Given the contemporaneous nature of the specimens used in this study, all of the RSVA and RSVB sequences contained the A.D and B.D lineage-defining *G*gene sequence duplications, respectively. Lineage A.D contains a 72 nt *G* gene duplication that emerged in 2011, and by 2017, its descendants had replaced all other lineages (15). Similarly, lineage B.D, first detected in 1999, contains a 60 nt *G*-gene duplication, and its descendants replaced all other lineages by 2009 (Fig. S3). The evolutionary impact of the duplications in these lineages is not well understood.

From a public health perspective, RSV genomes from different regions and time points provide important information on genetic changes that may affect viral pathogenicity, antigenicity, and vaccine efficacy. This knowledge can ultimately aid in the development of more effective vaccines and antiviral therapies. WGS provides a more complete understanding of the genetic diversity of RSV and permits a comprehensive genomic analysis of specific genomic regions associated with virulence, antigenicity, and drug resistance, which is important, given the fact that RSV does not employ a proofreading mechanism during replication (16). The assays described here generate near-complete genomes that can be readily characterized using both internal and publicly available tools, such as Nextclade and RSVsurver, to facilitate identification of emerging lineages, as well as the presence of biologically important or novel mutations (File S7). RSVsurver was used to visualize the aa changes identified for RSVA and RSVB (colored balls) among all available antigenic sites within the three-dimensional prefusion and postfusion structures of the RSV F glycoprotein in complex with AM22 (magenta) and Infant Antibody AD-19425 (green), respectively (Fig. S1 and S2). The prefusion conformation possesses all six major antigenic sites (Ø, I, II, III, IV, and V), of which only I, II, III, and IV are present in the postfusion conformation (44). Analysis using RSVsurver revealed that among the Canadian RSVA isolates, one isolate from SK collected in 2016 and three isolates collected from MB in 2023 possess the F:S377N mutation located in antigenic site III targeted by the RSVPreF3 vaccine, and this mutation might be in immunodominant sites for both RSVA and RSVB isolates (45). Moreover, one RSVA isolate collected from SK in 2016 contained the F:K272M mutation located in antigenic site II, which has been shown to impact the efficacy of palivizumab (46). Similarly, among the Canadian RSVB isolates, three mutations were identified, including F:S211N (*n* = 32), F:M206I (*n* = 3), and F:R209Q (*n* = 3) that are located in antigenic site Ø targeted by nirsevimab (Fig. 5; File S7). Thus, these isolates present an opportunity to conduct downstream antigenicity and antiviral testing to study the effect of both the well-characterized and rarer mutations observed across the data set.

In conclusion, the multiplex tiling PCR assays described in this study represent a convenient and inexpensive method for the rapid generation of near-complete RSV genomes from clinical specimens. These enhanced WGS methods can ultimately contribute to the advancement of RSV research, particularly in the areas of sequencing, diagnosis, and genomic surveillance. We have also demonstrated that the sequence data generated using our assays can be readily analyzed using downstream tools, including Nextclade for lineage assignment and RSVsurver for screening of important mutations, and can also be used to support epidemiological investigations within a genomic framework. These assays and subsequent genomic analyses offer potential for serving large-scale RSV genomic surveillance with enhanced efficiency and sensitivity, which will allow researchers to better monitor genomic variability in RSV and inform public health strategies for the development and usage of vaccines and antivirals.

## Supplementary Material

Reviewer comments

## Data Availability

All the complete respiratory syncytial virus genome sequences were deposited in the GISAID database, with the accession number being listed in File S7. The raw data are available under BioProject ID PRJNA1257940.

## SUPPLEMENTAL MATERIAL

File S1 spectrum.03142-24-s0001.txt hRSVA-tiling reference fasta.

File S2 spectrum.03142-24-s0002.txt hRSVA-tiling reference fasta fai.

File S3 spectrum.03142-24-s0003.txt hRSVA-Tiling scheme bed file.

File S4 spectrum.03142-24-s0004.txt hRSVB-tiling reference fasta.

File S5 spectrum.03142-24-s0005.txt hRSVB-tiling reference fasta fai.

File S6 spectrum.03142-24-s0006.txt RSVB-Tiling scheme bed file.

File S7 spectrum.03142-24-s0007.xlsx Metadata corresponding to the 176 RSVA and 123 RSVB genomes analyzed in this study.

File S8 spectrum.03142-24-s0008.txt R code used to generate the heatmap and co-phylogeny figures.

Supplemental figures spectrum.03142-24-s0009.pdf Fig. S1 to S3.

OPEN PEER REVIEW reviewer-comments.pdf An accounting of the reviewer comments and feedback.

ASM does not own the copyrights to Supplemental Material that may be linked to, or accessed through, an article. The authors have granted ASM a non-exclusive, world-wide license to publish the Supplemental Material files. Please contact the corresponding author directly for reuse.

## References

[1] Tan L, Coenjaerts FEJ, Houspie L, Viveen MC, van Bleek GM, Wiertz E, Martin DP, Lemey P. 2013. The comparative genomics of human respiratory syncytial virus subgroups A and B: genetic variability and molecular evolutionary dynamics. J Virol 87:8213–8226. doi:10.1128/JVI.03278-12 23698290 PMC3700225

[2] Hall CB, Simőes EAF, Anderson LJ. 2013. Clinical and epidemiologic features of respiratory syncytial virus. Curr Top Microbiol Immunol 372:39–57. doi:10.1007/978-3-642-38919-1_2 24362683

[3] Battles MB, McLellan JS. 2019. Respiratory syncytial virus entry and how to block it. Nat Rev Microbiol 17:233–245. doi:10.1038/s41579-019-0149-x 30723301 PMC7096974

[4] Tan L, Lemey P, Houspie L, Viveen MC, Jansen NJG, van Loon AM, Wiertz E, van Bleek GM, Martin DP, Coenjaerts FE. 2012. Genetic variability among complete human respiratory syncytial virus subgroup A genomes: bridging molecular evolutionary dynamics and epidemiology. PLoS One 7:e51439. doi:10.1371/journal.pone.0051439 23236501 PMC3517519

[5] Rebuffo-Scheer C, Bose M, He J, Khaja S, Ulatowski M, Beck ET, Fan J, Kumar S, Nelson MI, Henrickson KJ. 2011. Whole genome sequencing and evolutionary analysis of human respiratory syncytial virus A and B from Milwaukee, WI 1998-2010. PLoS One 6:e25468. doi:10.1371/journal.pone.0025468 21998661 PMC3188560

[6] Zhang N, Wang L, Deng X, Liang R, Su M, He C, Hu L, Su Y, Ren J, Yu F, Du L, Jiang S. 2020. Recent advances in the detection of respiratory virus infection in humans. J Med Virol 92:408–417. doi:10.1002/jmv.25674 31944312 PMC7166954

[7] Jha A, Jarvis H, Fraser C, Openshaw PJM. 2016. Respiratory syncytial virus. In Hui DS, Rossi GA, Johston SL (ed), SARS, MERS and other viral lung infections. European Respiratory Society, Sheffield, UK. 28742304

[8] Henrickson KJ, Hall CB. 2007. Diagnostic assays for respiratory syncytial virus disease. Pediatr Infect Dis J 26:S36–40. doi:10.1097/INF.0b013e318157da6f 18090198

[9] Zhu Y, Zembower TR, Metzger KE, Lei Z, Green SJ, Qi C. 2017. Investigation of respiratory syncytial virus outbreak on an adult stem cell transplant unit by use of whole-genome sequencing. J Clin Microbiol 55:2956–2963. doi:10.1128/JCM.00360-17 28747373 PMC5625381

[10] Johnson PCD, Hägglund S, Näslund K, Meyer G, Taylor G, Orton RJ, Zohari S, Haydon DT, Valarcher JF. 2022. Evaluating the potential of whole-genome sequencing for tracing transmission routes in experimental infections and natural outbreaks of bovine respiratory syncytial virus. Vet Res 53:107. doi:10.1186/s13567-022-01127-9 36510312 PMC9746130

[11] Lin G-L, Golubchik T, Drysdale S, O’Connor D, Jefferies K, Brown A, de Cesare M, Bonsall D, Ansari MA, Aerssens J, Bont L, Openshaw P, Martinón-Torres F, Bowden R, Pollard AJ, RESCEU Investigators. 2020. Simultaneous viral whole-genome sequencing and differential expression profiling in respiratory syncytial virus infection of infants. J Infect Dis 222:S666–S671. doi:10.1093/infdis/jiaa448 32702120

[12] Ferdinand AS, Kelaher M, Lane CR, da Silva AG, Sherry NL, Ballard SA, Andersson P, Hoang T, Denholm JT, Easton M, Howden BP, Williamson DA. 2021. An implementation science approach to evaluating pathogen whole genome sequencing in public health. Genome Med 13:121. doi:10.1186/s13073-021-00934-7 34321076 PMC8317677

[13] Panatto D, Domnich A, Lai PL, Ogliastro M, Bruzzone B, Galli C, Stefanelli F, Pariani E, Orsi A, Icardi G. 2023. Epidemiology and molecular characteristics of respiratory syncytial virus (RSV) among italian community-dwelling adults, 2021/22 season. BMC Infect Dis 23:134. doi:10.1186/s12879-023-08100-7 36882698 PMC9990006

[14] Aksamentov I, Roemer C, Hodcroft EB, Neher RA. 2021. Nextclade: clade assignment, mutation calling and quality control for viral genomes. J Open Source Software 6:3773. doi:10.21105/joss.03773

[15] Goya S, Ruis C, Neher RA, Meijer A, Aziz A, Hinrichs AS, von Gottberg A, Roemer C, Amoako DG, Acuña D, *et al*.*.* 2024. Standardized phylogenetic classification of human respiratory syncytial virus below the subgroup level. Emerg Infect Dis 30:1631–1641. doi:10.3201/eid3008.240209 39043393 PMC11286072

[16] Griffiths C, Drews SJ, Marchant DJ. 2017. Respiratory syncytial virus: infection, detection, and new options for prevention and treatment. Clin Microbiol Rev 30:277–319. doi:10.1128/CMR.00010-16 27903593 PMC5217795

[17] Schobel SA, Stucker KM, Moore ML, Anderson LJ, Larkin EK, Shankar J, Bera J, Puri V, Shilts MH, Rosas-Salazar C, Halpin RA, Fedorova N, Shrivastava S, Stockwell TB, Peebles RS, Hartert TV, Das SR. 2016. Respiratory syncytial virus whole-genome sequencing identifies convergent evolution of sequence duplication in the C-terminus of the G gene. Sci Rep 6:26311. doi:10.1038/srep26311 27212633 PMC4876326

[18] Schaerlaekens S, Jacobs L, Stobbelaar K, Cos P, Delputte P. 2024. All eyes on the prefusion-stabilized F construct, but are we missing the potential of alternative targets for respiratory syncytial virus vaccine design? Vaccines (Basel) 12:97. doi:10.3390/vaccines12010097 38250910 PMC10819635

[19] Mazur NI, Terstappen J, Baral R, Bardají A, Beutels P, Buchholz UJ, Cohen C, Crowe JE Jr, Cutland CL, Eckert L, *et al*.*.* 2023. Respiratory syncytial virus prevention within reach: the vaccine and monoclonal antibody landscape. Lancet Infect Dis 23:e2–e21. doi:10.1016/S1473-3099(22)00291-2 35952703 PMC9896921

[20] Langedijk AC, Bont LJ. 2023. Respiratory syncytial virus infection and novel interventions. Nat Rev Microbiol 21:734–749. doi:10.1038/s41579-023-00919-w 37438492

[21] Health Canada. 2023. Summary basis of decision (SBD) for Arexvy. Office of regulatory affairs BaRDDO-B. Drug and Health Product Portal. Health Canada. https://dhpp.hpfb-dgpsa.ca/review-documents/resource/SBD1701180125532.

[22] Canada Go. 2024. Respiratory syncytial virus (RSV) vaccines: Canadian immunization guide

[23] Ko S, Park S, Sohn MH, Jo M, Ko BJ, Na J-H, Yoo H, Jeong AL, Ha K, Woo JR, Lim C, Shin JH, Lee D, Choi S-Y, Jung ST. 2022. An Fc variant with two mutations confers prolonged serum half-life and enhanced effector functions on IgG antibodies. Exp Mol Med 54:1850–1861. doi:10.1038/s12276-022-00870-5 36319752 PMC9628495

[24] Domachowske JB, Khan AA, Esser MT, Jensen K, Takas T, Villafana T, Dubovsky F, Griffin MP. 2018. Safety, tolerability and pharmacokinetics of MEDI8897, an extended half-life single-dose respiratory syncytial virus prefusion F-targeting monoclonal antibody administered as a single dose to healthy preterm infants. Pediatr Infect Dis J 37:886–892. doi:10.1097/INF.0000000000001916 29373476 PMC6133204

[25] Agoti CN, Otieno JR, Munywoki PK, Mwihuri AG, Cane PA, Nokes DJ, Kellam P, Cotten M. 2015. Local evolutionary patterns of human respiratory syncytial virus derived from whole-genome sequencing. J Virol 89:3444–3454. doi:10.1128/JVI.03391-14 25609811 PMC4403408

[26] Pangesti KNA, Ansari HR, Bayoumi A, Kesson AM, Hill-Cawthorne GA, Abd El Ghany M. 2023. Genomic characterization of respiratory syncytial virus genotypes circulating in the paediatric population of Sydney, NSW, Australia. Microb Genom 9:001095. doi:10.1099/mgen.0.001095 37656160 PMC10569731

[27] Holland LA, Holland SC, Smith MF, Leonard VR, Murugan V, Nordstrom L, Mulrow M, Salgado R, White M, Lim ES. 2023. Genomic sequencing surveillance to identify respiratory syncytial virus mutations, Arizona, USA. Emerg Infect Dis 29:2380–2382. doi:10.3201/eid2911.230836 37705075 PMC10617361

[28] Goya S, Sereewit J, Pfalmer D, Nguyen TV, Bakhash S, Sobolik EB, Greninger AL. 2023. Genomic characterization of respiratory syncytial virus during 2022-23 outbreak, Washington, USA. Emerg Infect Dis 29:865–868. doi:10.3201/eid2904.221834 36878012 PMC10045680

[29] Lin G-L, Drysdale SB, Snape MD, O’Connor D, Brown A, MacIntyre-Cockett G, Mellado-Gomez E, de Cesare M, Ansari MA, Bonsall D, Bray JE, Jolley KA, Bowden R, Aerssens J, Bont L, Openshaw PJM, Martinon-Torres F, Nair H, Golubchik T, Pollard AJ, RESCEU Consortium. 2024. Targeted metagenomics reveals association between severity and pathogen co-detection in infants with respiratory syncytial virus. Nat Commun 15:2379. doi:10.1038/s41467-024-46648-3 38493135 PMC10944482

[30] Quick J, Grubaugh ND, Pullan ST, Claro IM, Smith AD, Gangavarapu K, Oliveira G, Robles-Sikisaka R, Rogers TF, Beutler NA, *et al*.*.* 2017. Multiplex PCR method for MinION and Illumina sequencing of Zika and other virus genomes directly from clinical samples. Nat Protoc 12:1261–1276. doi:10.1038/nprot.2017.066 28538739 PMC5902022

[31] Talts T, Mosscrop LG, Williams D, Tregoning JS, Paulo W, Kohli A, Williams TC, Hoschler K, Ellis J, Lusignan S de, Zambon M. 2024. Robust and sensitive amplicon-based whole-genome sequencing assay of respiratory syncytial virus subtype A and B. Microbiol Spectr 12:e03067-23. doi:10.1128/spectrum.03067-23 38411056 PMC10986592

[32] Wong H, Sjaarda CP, Rand B, Roberts D, Tozer K, Fattouh R, Kozak R, Sheth PM. 2025. The molecular epidemiology of respiratory syncytial virus in Ontario, Canada from 2022-2024 using a custom whole genome sequencing assay and analytics package. J Clin Virol 176:105759. doi:10.1016/j.jcv.2024.105759 39721564

[33] Li W, Godzik A. 2006. Cd-hit: a fast program for clustering and comparing large sets of protein or nucleotide sequences. Bioinformatics 22:1658–1659. doi:10.1093/bioinformatics/btl158 16731699

[34] Katoh K, Misawa K, Kuma K, Miyata T. 2002. MAFFT: a novel method for rapid multiple sequence alignment based on fast Fourier transform. Nucleic Acids Res 30:3059–3066. doi:10.1093/nar/gkf436 12136088 PMC135756

[35] Word Health Organization. 2019. WHO strategy for global respiratory syncytial virus surveillance project based on the influenza platform. https://cdn.who.int/media/docs/default-source/influenza/rsv-surveillance/who-rsv-surveillance-strategy-phase-26mar2021.-final.pdf?sfvrsn=d8b1c36a_9.

[36] Goya S, Galiano M, Nauwelaers I, Trento A, Openshaw PJ, Mistchenko AS, Zambon M, Viegas M. 2020. Toward unified molecular surveillance of RSV: a proposal for genotype definition. Influenza Resp Viruses 14:274–285. doi:10.1111/irv.12715 PMC7182609 32022426

[37] Price MN, Dehal PS, Arkin AP. 2009. FastTree: computing large minimum evolution trees with profiles instead of a distance matrix. Mol Biol Evol 26:1641–1650. doi:10.1093/molbev/msp077 19377059 PMC2693737

[38] Price MN, Dehal PS, Arkin AP. 2010. FastTree 2 – approximately maximum-likelihood trees for large alignments. PLoS One 5:e9490. doi:10.1371/journal.pone.0009490 20224823 PMC2835736

[39] Letunic I, Bork P. 2021. Interactive Tree Of Life (iTOL) v5: an online tool for phylogenetic tree display and annotation. Nucleic Acids Res 49:W293–W296. doi:10.1093/nar/gkab301 33885785 PMC8265157

[40] Hotard AL, Lee S, Currier MG, Crowe JE, Sakamoto K, Newcomb DC, Peebles RS, Plemper RK, Moore ML. 2015. Identification of residues in the human respiratory syncytial virus fusion protein that modulate fusion activity and pathogenesis. J Virol 89:512–522. doi:10.1128/JVI.02472-14 25339762 PMC4301159

[41] Li K, Shrivastava S, Brownley A, Katzel D, Bera J, Nguyen AT, Thovarai V, Halpin R, Stockwell TB. 2012. Automated degenerate PCR primer design for high-throughput sequencing improves efficiency of viral sequencing. Virol J 9:261. doi:10.1186/1743-422X-9-261 23131097 PMC3548747

[42] Isabel S, Eshaghi A, Duvvuri VR, Gubbay JB, Cronin K, Li A, Hasso M, Clark ST, Hopkins JP, Patel SN, Braukmann TWA. 2024. Targeted amplification-based whole genome sequencing of Monkeypox virus in clinical specimens. Microbiol Spectr 12:e02979-23. doi:10.1128/spectrum.02979-23 38047694 PMC10783113

[43] Lambisia AW, Mohammed KS, Makori TO, Ndwiga L, Mburu MW, Morobe JM, Moraa EO, Musyoki J, Murunga N, Mwangi JN, Nokes DJ, Agoti CN, Ochola-Oyier LI, Githinji G. 2022. Optimization of the SARS-CoV-2 ARTIC network V4 primers and whole genome sequencing protocol. Front Med (Lausanne) 9:836728. doi:10.3389/fmed.2022.836728 35252269 PMC8891481

[44] Tang A, Chen Z, Cox KS, Su HP, Callahan C, Fridman A, Zhang L, Patel SB, Cejas PJ, Swoyer R, Touch S, Citron MP, Govindarajan D, Luo B, Eddins M, Reid JC, Soisson SM, Galli J, Wang D, Wen Z, Heidecker GJ, Casimiro DR, DiStefano DJ, Vora KA. 2019. A potent broadly neutralizing human RSV antibody targets conserved site IV of the fusion glycoprotein. Nat Commun 10:4153. doi:10.1038/s41467-019-12137-1 31515478 PMC6742648

[45] Lu B, Liu H, Tabor DE, Tovchigrechko A, Qi Y, Ruzin A, Esser MT, Jin H. 2019. Emergence of new antigenic epitopes in the glycoproteins of human respiratory syncytial virus collected from a US surveillance study, 2015–17. Sci Rep 9:3898. doi:10.1038/s41598-019-40387-y 30846850 PMC6405860

[46] Zhu Q, McAuliffe JM, Patel NK, Palmer-Hill FJ, Yang C, Liang B, Su L, Zhu W, Wachter L, Wilson S, MacGill RS, Krishnan S, McCarthy MP, Losonsky GA, Suzich JA. 2011. Analysis of respiratory syncytial virus preclinical and clinical variants resistant to neutralization by monoclonal antibodies palivizumab and/or motavizumab. J Infect Dis 203:674–682. doi:10.1093/infdis/jiq100 21208913 PMC3072724

