# Supplementary material for: Whole genome sequencing and phylogenetic classification accelerate the implementation of respiratory syncytial virus genomic surveillance in Canada: a pilot study
Source: Microbiol Spectr. 2025 Sep 3;13(10):e03142-24. doi: 10.1128/spectrum.03142-24 (PMC12502720; doi:10.1128/spectrum.03142-24)
Supplement: Supplemental figures — Fig. S1 to S3. [file spectrum.03142-24-s0009.pdf]

SUPPLEMENTAL FIG 1

A Prefusion

B Postfusion

176 RSVA

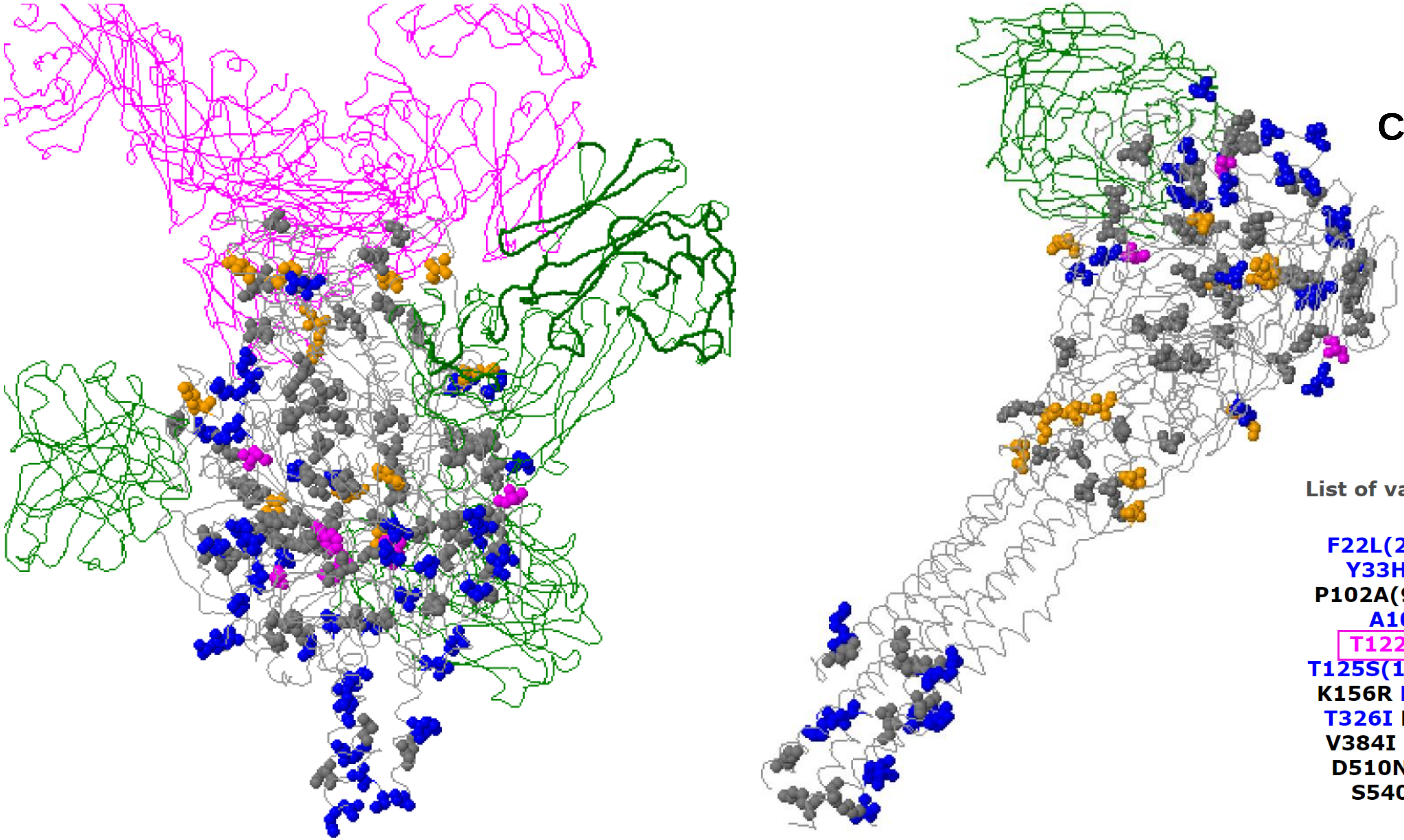

C

T122N: RV00332; RV00334

T122A : RV00279-00284; RV00287-00290; RV00292-00294; RV00312-00315; RV00317; RV00333; RV00335-00337; RV00339; RV00342-00344; RV01412; RV01414-01415

K272M: RV00295

List of variations displayed in structure (nearest residue if in loop/termini region)

F22L(27) F22I(27) F22S(27) S24F(27) G25S(27) T29I
Y33H S35A L45F N67T K77R I79M N88S T100S(98)
P102A(98) A103T(98) T103S(98) T103A(98) N104S(98)
A107V(98) M115V(98) N116S(98) T122N(137)
T122A(137) A122T(137) K124N(137) T125N(137)
T125S(137) V127A(137) V127I(137) V139G V144I V152I
K156R N165D L178V S213R N240K K272M S276N T324I
T326I L334I E356D N371H P376S S377N I379V N380K
V384I I384T P389L P389S V402I M447V S451T Q462K
D510N N515S N515T N515H A518V(517) S521T(517)
S540L(517) A540S(517) A543V(517) V544A(517)
L547F(517) R553K(517) V557I(517)

SUPPLEMENTAL FIG.1 Presence of all amino acid mutations as identified by RSVsurver from the 176 RSVA sequences used in this study contextualized within the 3D structure of the RSV F glycroprotein. (A) prefusion RSV F glycoprotein (PDB: 6apd, X-ray 4.1 Angstrom) in complex with AM22 (magenta ribbon) & Infant Antibody ADI-19425 (green ribbon). (B) postfusion RSV F glycoprotein (PDB: 6apb, X-ray 3.0 Angstrom) in complex with Infant Antibody ADI-14359 (green ribbon). The mutations are color-coded by RSVsurver according to their known or predicted biological effect significance. When there are no known effects for the mutation, the mutation will appear in black colored font and assigned interestlevel 0 (least significant). Mutations occurring at a site of interaction will appear in blue colored font and assigned interestlevel 1 (moderately significant). If the mutation occurs at a site known to be involved in drug-binding or alters host-cell specificity, it will appear in orange and assigned interestlevel 2 (significant). Mutations will also appear in orange and assigned interest level 2 when its equivalent site is known to result in antigenic shifts or causes mild drug resistance. Mutations that create or remove a potential glycosylation site are colored magenta and assigned interestlevel 2.

SUPPLEMENTAL FIG. 2

A Prefusion

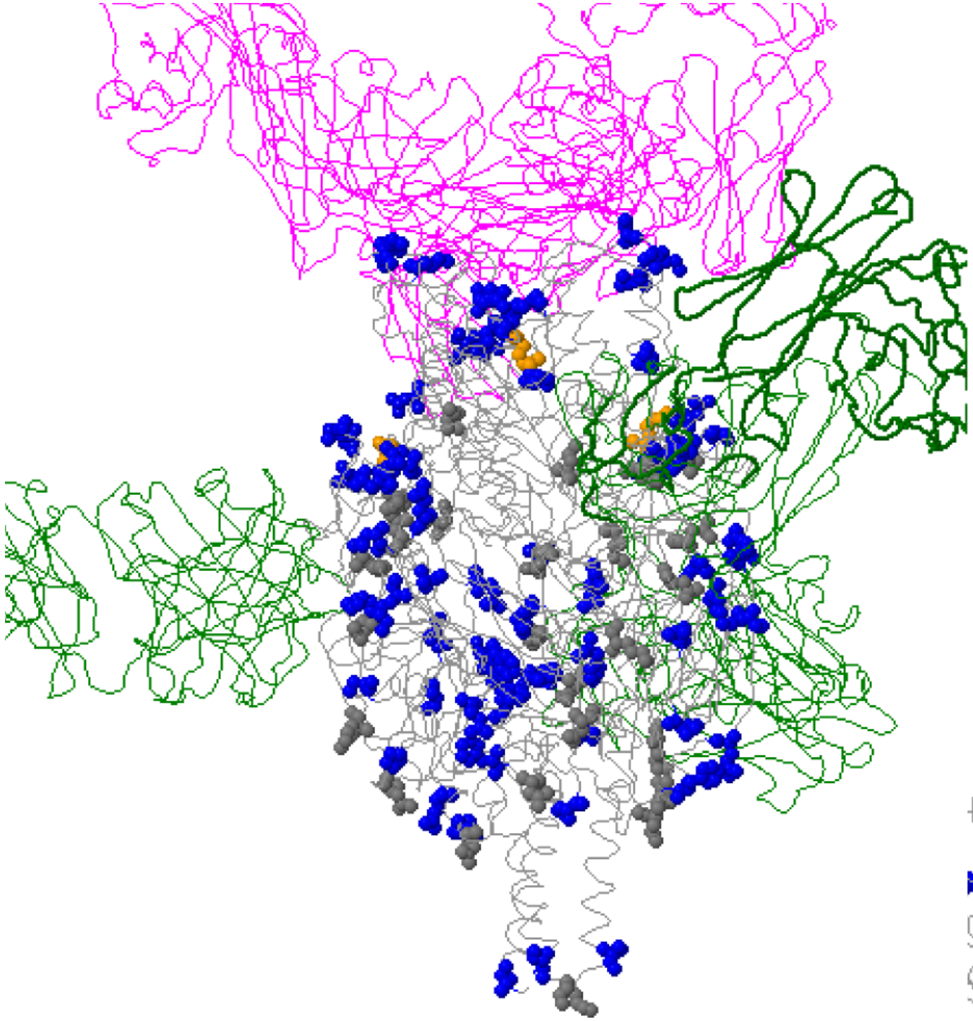

B Postfusion

123 RSVB

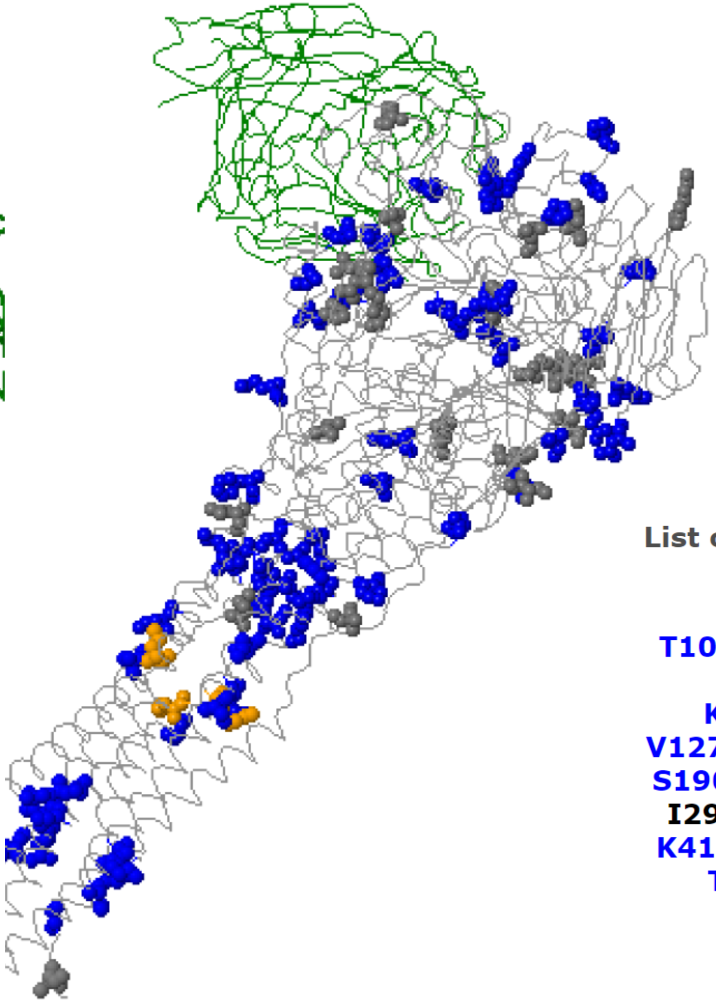

C R191K: RV00297; RV00298; VR-1794; VR-1803; VR-955

List of variations displayed in structure (nearest residue if in loop/termini region)

Y21C(27) T23A(27) R42K L45F L45W N99S(98) T100I(98) V103A(98) V103I(98) N104S(98) M115I(98) N116D(98) Y117H(98) T121A(137) K123G(137) K123N(137) N124S(137) L125P(137) V127A(137) V127I(137) I129T(137) K132R(137) N169S Q172L L173S S190N R191K N197D M206I R209Q S211N S276N N277H I291V L303I P312H N325S D344E D356E S377N S389P K419N K419E E463D N466S E472K Y477H D479N H514R T518A(517) A529V(517) A529T(517) I542V(517) I544V(517) L548F(517) K551R(517)

SUPPLEMENTAL FIG. 2 Presence of all amino acid mutations as identified by RSVsurver from the 123 RSVB sequences used in this study contextualized within the 3D structure of the RSV F glycroprotein. (A) prefusion RSV F glycoprotein (PDB: 6apd, X-ray 4.1 Angstrom) in complex with AM22 (magenta ribbon) & Infant Antibody ADI-19425 (green ribbon). (B) postfusion RSV F glycoprotein (PDB: 6apb, X-ray 3.0 Angstrom) in complex with Infant Antibody ADI-14359 (green ribbon). The mutations are color-coded by RSVsurver according to their known or predicted biological effect significance. When there are no known effects for the mutation, the mutation will appear in black colored font and assigned interestlevel 0 (least significant). Mutations occurring at a site of interaction will appear in blue colored font and assigned interestlevel 1 (moderately significant). If the mutation occurs at a site known to be involved in drug-binding or alters host-cell specificity, it will appear in orange and assigned interestlevel 2 (significant). Mutations will also appear in orange and assigned interest level 2 when its equivalent site is known to result in antigenic shifts or causes mild drug resistance. Mutations that create or remove a potential glycosylation site are colored magenta and assigned interestlevel 2.

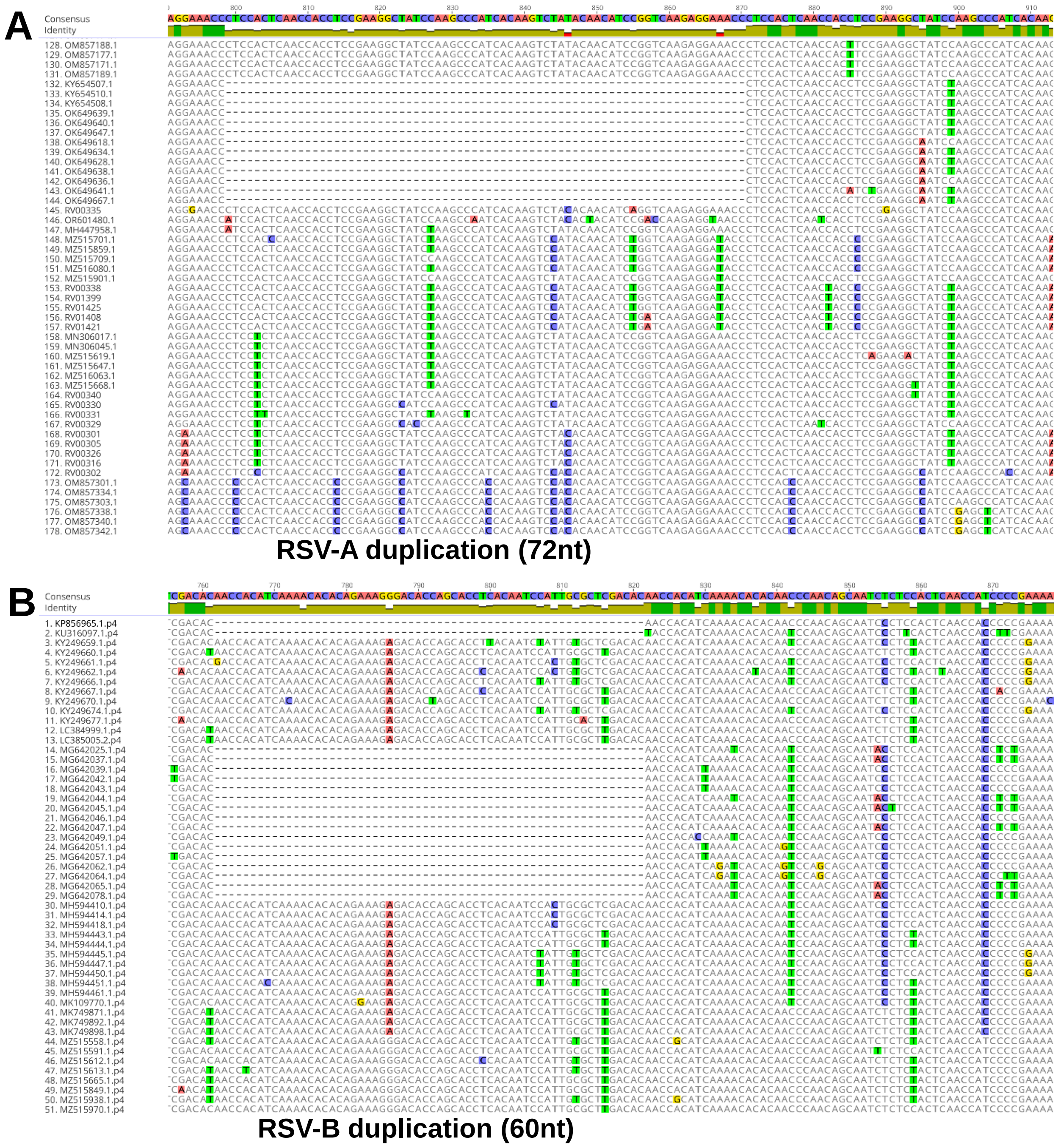

SUPPLEMENTAL FIG. 3 RSV lineage-defining G gene duplication events.

(A) Representative sequences containing the A.D lineage defining 72-nt duplication.

(B) Representative sequences containing the B.D lineage-defining 60-nt duplication.
